# Author Correction: Upscaled production of an ultramicroporous anion-exchange membrane enables long-term operation in electrochemical energy devices

**DOI:** 10.1038/s41467-023-40059-6

**Published:** 2023-07-25

**Authors:** Wanjie Song, Kang Peng, Wei Xu, Xiang Liu, Huaqing Zhang, Xian Liang, Bangjiao Ye, Hongjun Zhang, Zhengjin Yang, Liang Wu, Xiaolin Ge, Tongwen Xu

**Affiliations:** 1 CAS Key Laboratory of Soft Matter Chemistry, Collaborative Innovation Centre of Chemistry for Energy Materials, School of Chemistry and Material Science, University of Science and Technology of China, Hefei, 230026 PR China; 2 State Key Laboratory of Particle Detectionand Electronics, University of Science and Technology of China, Hefei, 230026 PR China

**Keywords:** Fuel cells, Polymers, Chemical engineering, Fuel cells, Polymer chemistry

Correction to: *Nature Communications* 10.1038/s41467-023-38350-7, published online 12 May 2023

In Fig. 4d “Energy efficiency” data were incorrectly plotted and the arrows to indicate the corresponding y-axes were absent; the figure should have appeared as shown below.


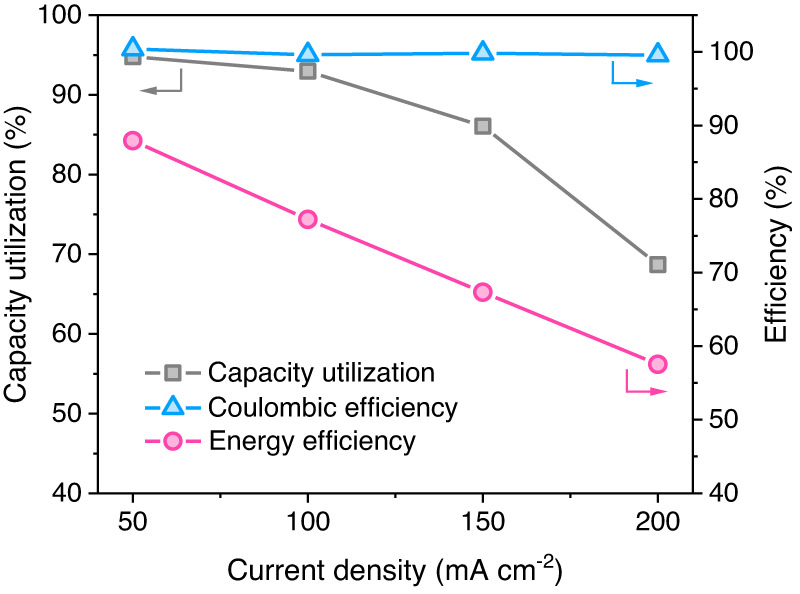


Additionally, in Fig. 6a the arrows indicating the directionality of species fluxes across the membrane were inadvertently reversed; the figure should have appeared as shown below.


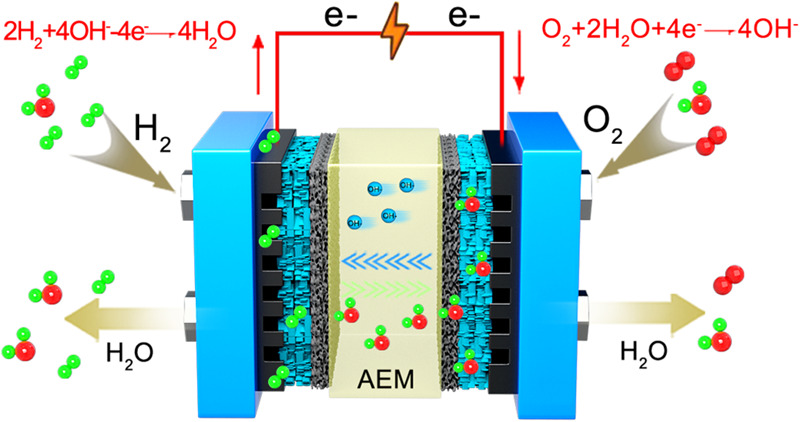


The original article has been corrected.

