# Supplementary material for: Upscaled production of an ultramicroporous anion-exchange membrane enables long-term operation in electrochemical energy devices
Source: Nat Commun. 2023 May 12;14:2732. doi: 10.1038/s41467-023-38350-7 (PMC10175247; doi:10.1038/s41467-023-38350-7)
Supplement: Supplementary file 1 — Supplementary Information [file 41467_2023_38350_MOESM1_ESM.pdf]

Upscaled production of an ultramicroporous anion-exchange membrane enables long-term operation in electrochemical energy devices

Wanjie Song[1], Kang Peng[1], Wei Xu[2], Xiang Liu[1], Huaqing Zhang[1], Xian Liang[1], Bangjiao Ye[2], Hongjun Zhang[2], Zhengjin Yang[1], Liang Wu[1,*], Xiaolin Ge[1,*], Tongwen Xu[1,*]

[1] CAS Key Laboratory of Soft Matter Chemistry, Collaborative Innovation Centre of Chemistry for Energy Materials, School of Chemistry and Material Science, University of Science and Technology of China, Hefei 230026, P.R. China

[2] State Key Laboratory of Particle Detectionand Electronics, University of Science and Technology of China, Hefei 230026, P.R. China

*Corresponding Author: twxu@ustc.edu.cn, gexl@ustc.edu.cn, liangwu8@ustc.edu.cn

These authors contributed equally: Author: Wanjie Song, Author: Kang Peng

1. AEM fabrication procedures and ^{1}H NMR spectra confirmation.

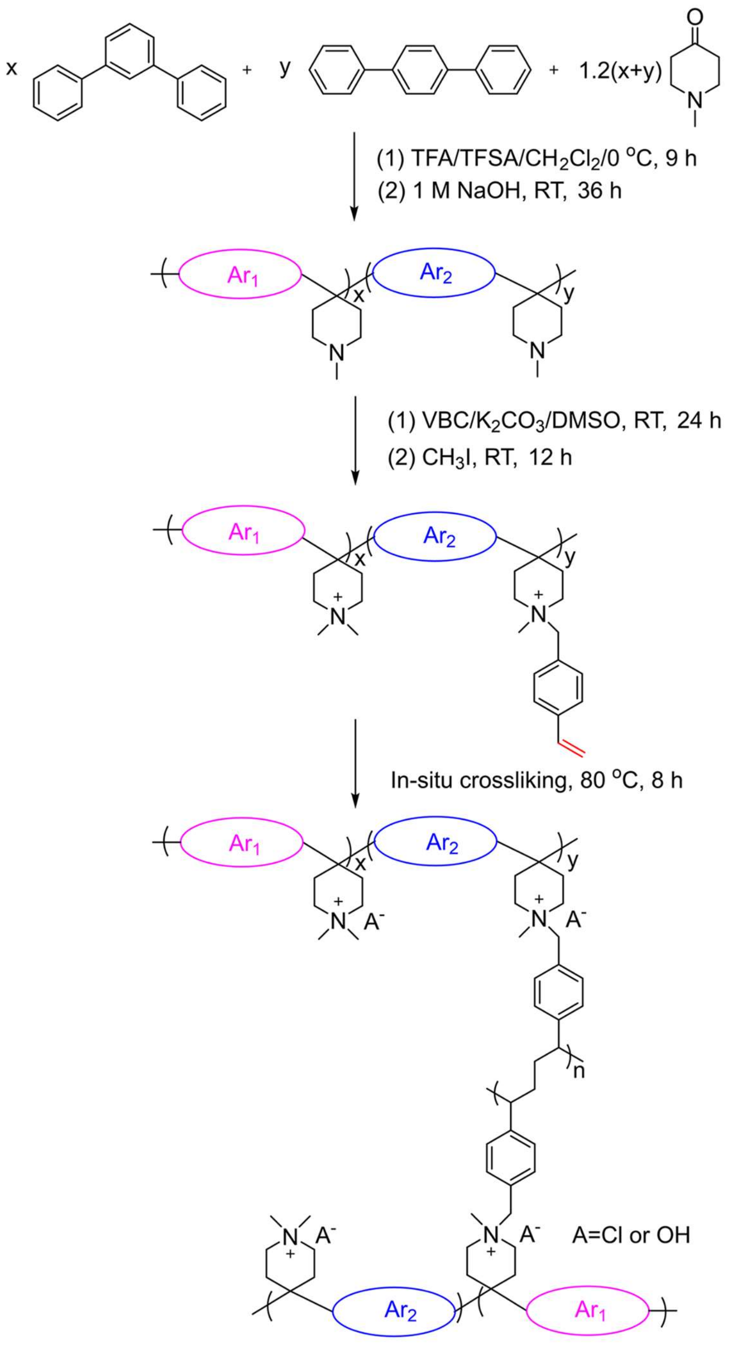

Supplementary Fig. 1 | Synthesis routes of polymer MTCP-x.

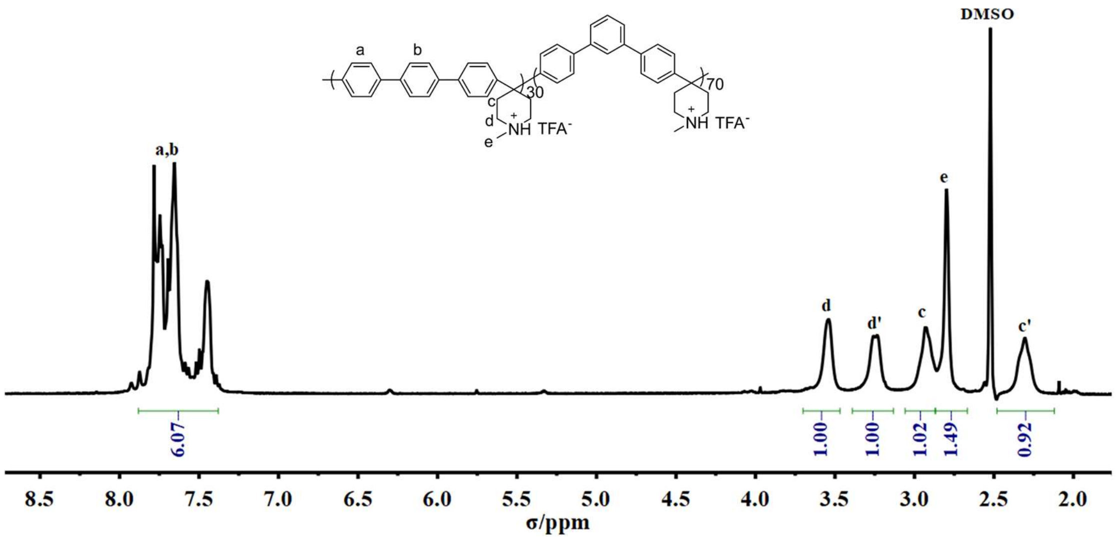

Supplementary Fig. 2a | ^{1}H NMR spectrum of MTP-30 in DMSO-d6 with 10% TFA as co-solvent to eliminate the effect of H_2O at 3.34 ppm.

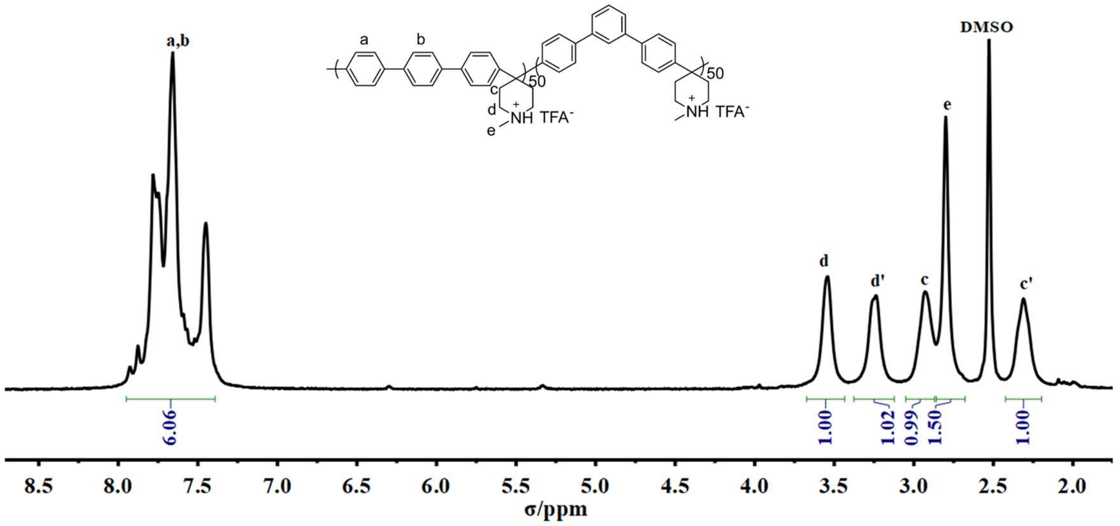

Supplementary Fig. 2b | ^{1}H NMR spectrum of MTP-50 in DMSO-d6 with 10% TFA as co-solvent to eliminate the effect of H_2O at 3.34 ppm.

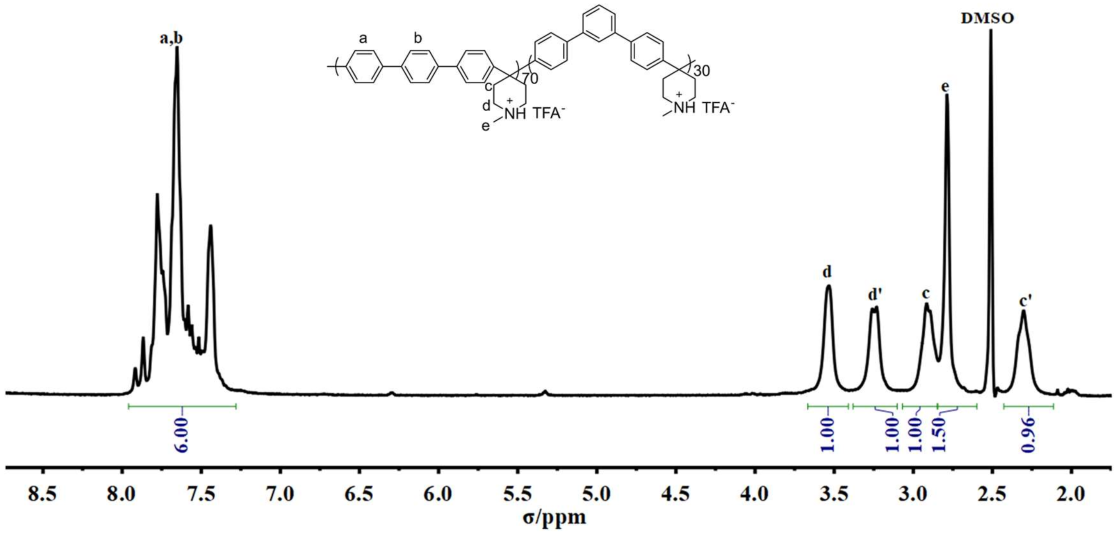

Supplementary Fig. 2c | ^{1}H NMR spectrum of MTP-70 in DMSO-d6 with 10% TFA

as co-solvent to eliminate the effect of H_2O at 3.34 ppm.

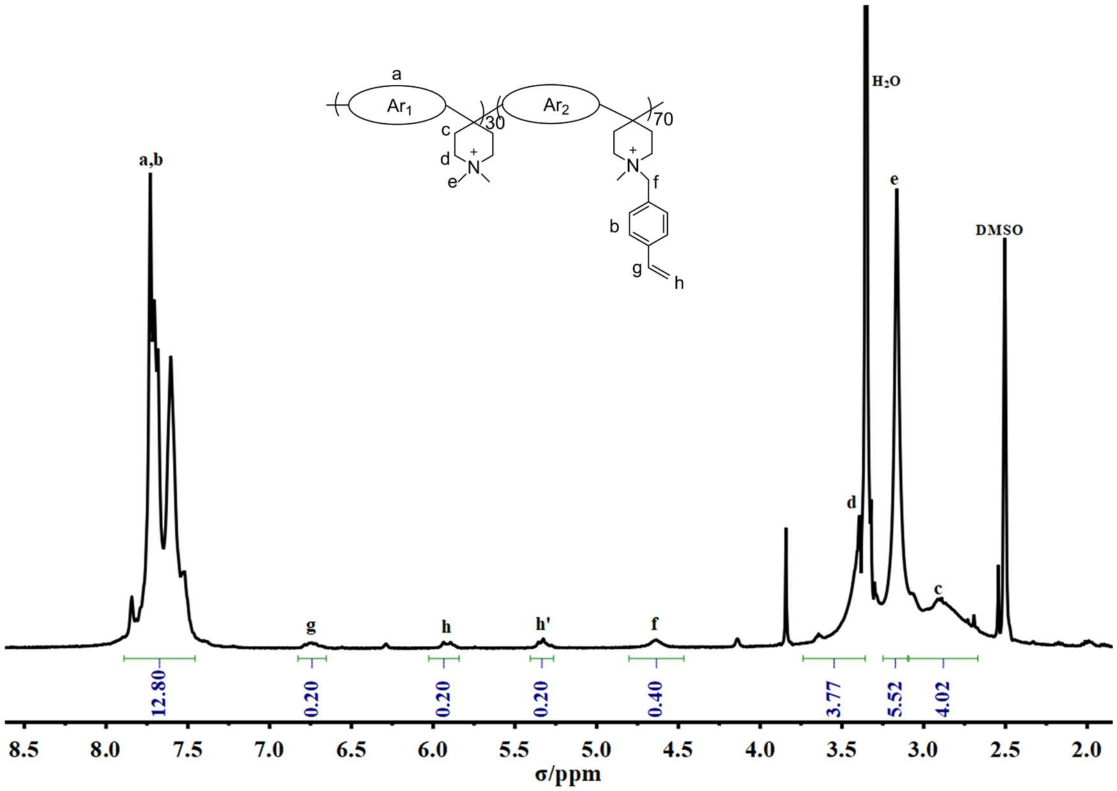

Supplementary Fig. 2d | ^{1}H NMR spectrum of MTCP-30 in DMSO-d6.

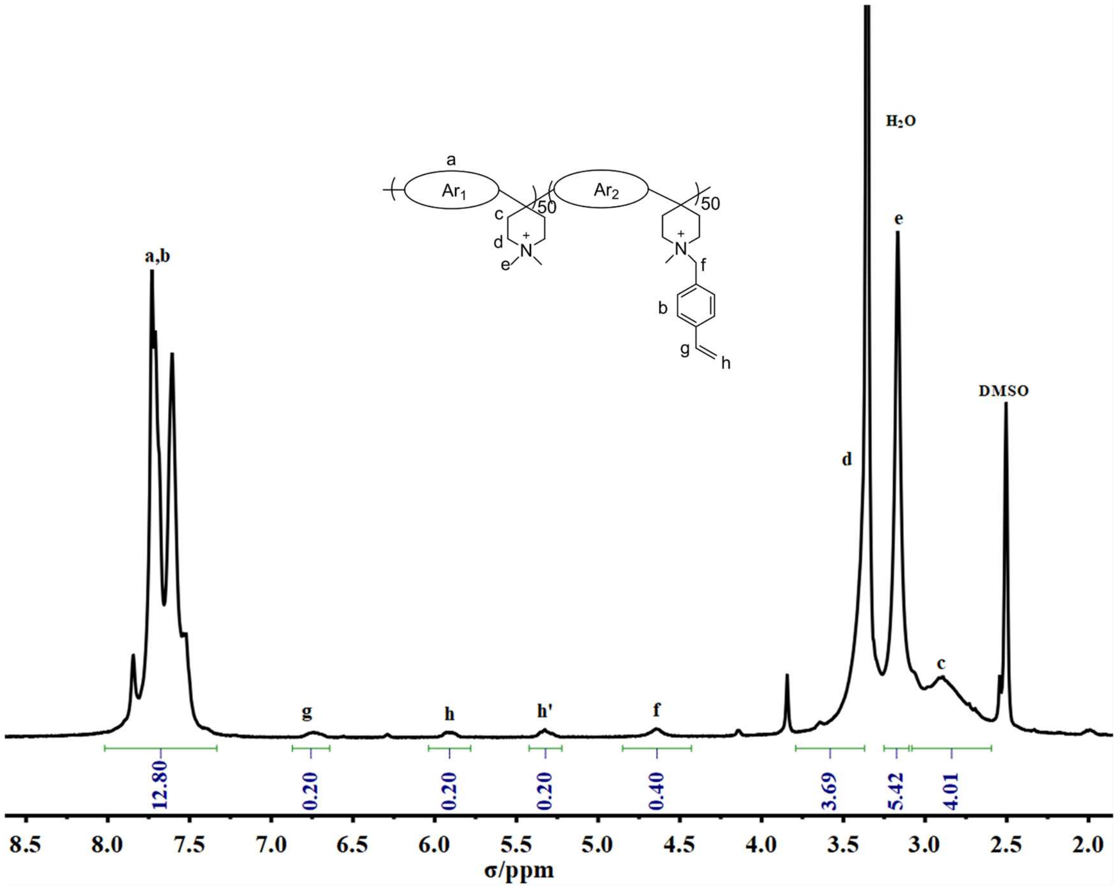

Supplementary Fig. 2e | ^{1}H NMR spectrum of MTCP-50 in DMSO-d6.

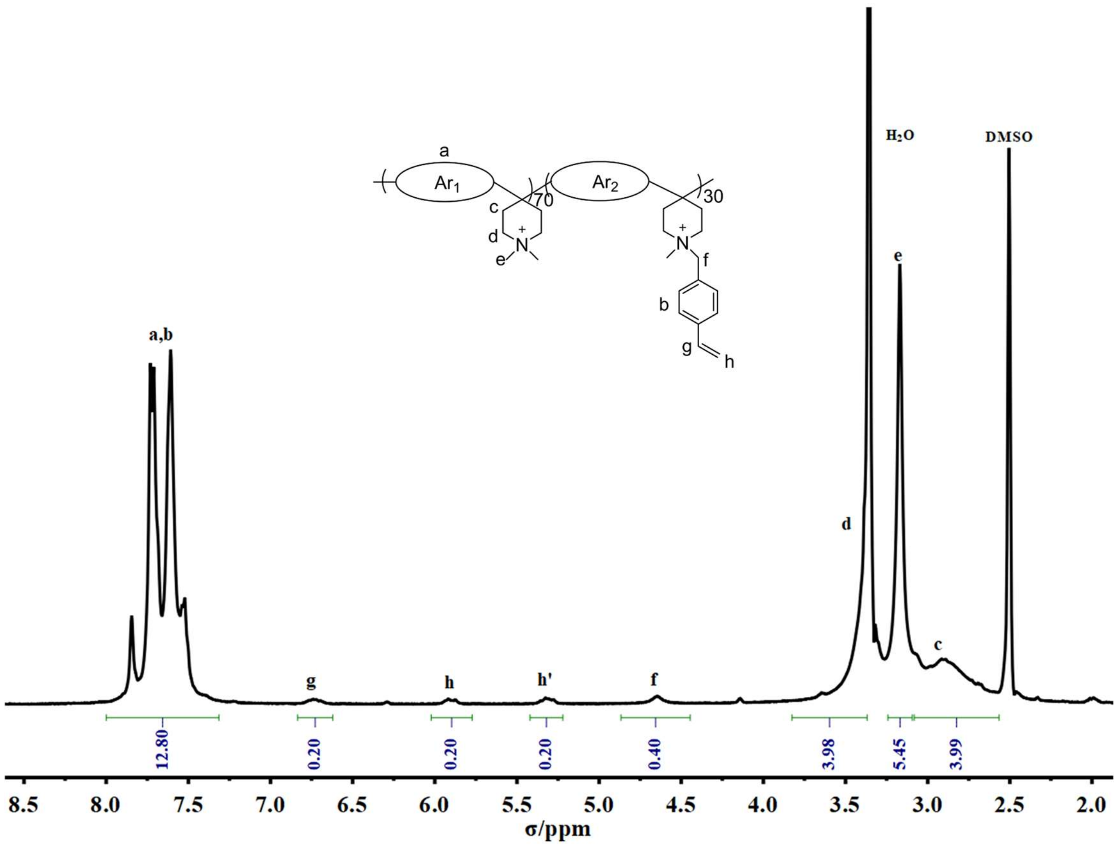

Supplementary Fig. 2f | ^{1}H NMR spectrum of MTCP-70 in DMSO-d6.

2. Experiment details

Permeability measurement. The permeability of TEMPTMA and MV across the MTCP-50 was measured using an H-cell. The donating side was filled with 2 M of electrolyte in DI water, whereas the receiving side comprised the same volume of NaCl aqueous solution (2.5 M for TEMPTMA while 3 M for MV), which equalized the ionic strengths and minimized the osmotic pressure effect. Both the donating and the receiving compartments were continuously stirred. At different time intervals, a certain volume of sample was collected from the receiving side. After dilution, it was characterized by UV-visible spectrophotometry, and the concentration was calculated according to the calibration curve. The permeability (P) was then calculated based on the slope of a linear regression of concentration in the receiving compartment vs. time, using Fick's law as embodied in the following equation:

$$P=\frac{\ln(1-\frac{2C_r}{C_0})(-\frac{Vl}{2A})}{t}$$

Where Cr is the concentration measured at the receiving reservoir, C_0 is the active species concentration in the donating reservoir (2 M), V is the volume of the receiving side (20 mL), l is the membrane thickness (54 μm), A is the membrane area (1.89 cm^2), and t is the time.

3. Supplementary figures

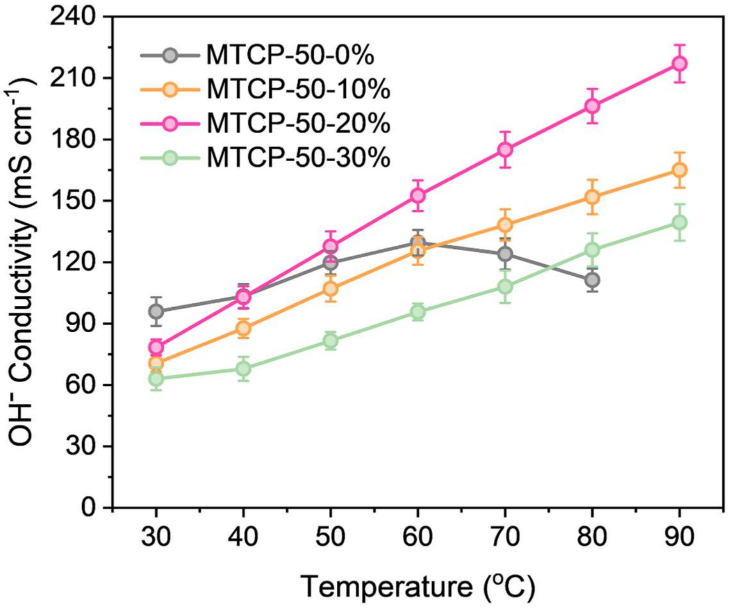

Supplementary Fig. 3 | Temperature-dependent OH^- conductivity of MTCP-50 with different VBC grafting ratios. The error bars represent the standard deviation of OH^- conductivity.

Notes: MTCP-x AEMs have a high IEC value when direct quaternary amination by methyl iodide. The excessive water absorption dilutes the concentration of cationic groups within the membrane, especially at high temperatures. Hence results in a decrease in ion conductivity, mechanical loss et al. For example, the non-crosslinked MTCP-50-0% with high IEC value reaches high conductivity at 30 °C, but subsequently shows a decrease in OH^- conductivity when temperature above 60 °C. This poor dimensional stability is a challenge for application. In the case of polymers with high IEC, lightly cross-linking is an effective method to reduce the water uptake and swelling ratio and benefits the balance of high conductivity and water uptake without sacrificing conductivity. But, more dense cross-linking network within AEMs, results in poor ion mobility, water diffusivity, and mechanical properties. Therefore, we optimized the cross-linking degree by monitoring OH^- conductivity changes. The experimental results show that the AEM has excellent high-temperature tolerance and delivers a higher OH^- conductivity level when the cross-linking degree is 20%.

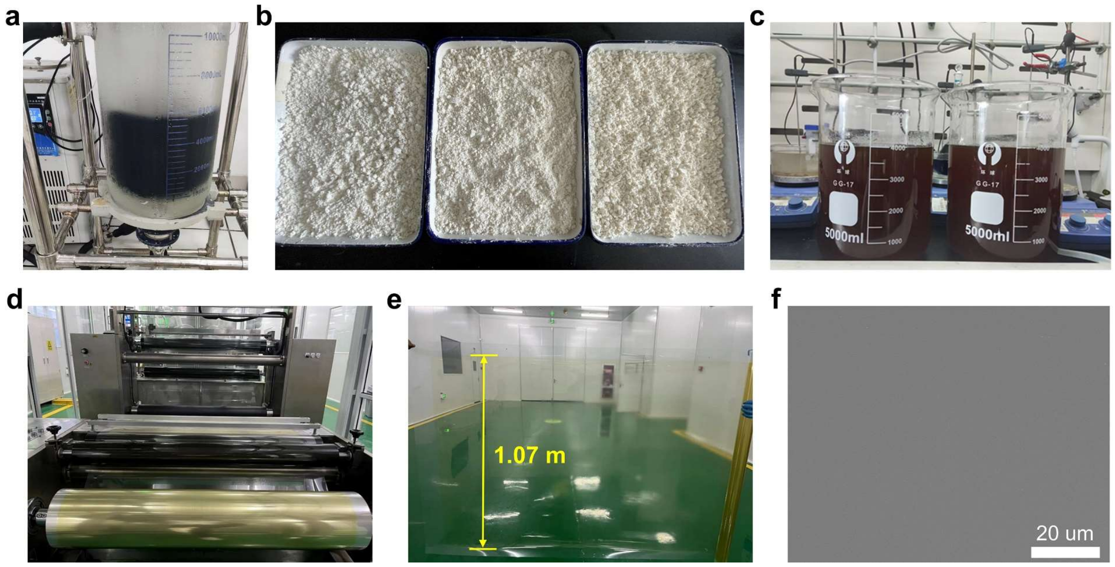

Supplementary Fig. 4 | Pilot-scale synthesis and roll-to-roll manufacturing of MTCP-50 AEM. a, Photo images of a reactor for kg-scale MTCP-50 polymer synthesis. **b,** Polymer powder, **c,** Polymer casting solution. **d,** Roll-to-roll membrane casting machine. **e,** Polymer membrane with a width > 1000 mm. **f,** Surface morphology of MTCP-50 AEM.

Notes: The preparation of membrane products includes the synthesis of polymer electrolyte materials and roll-to-roll production of membrane casting solutions. As shown in **Supplementary Fig. 4**, we synthesized kg-scale polymer with a 10 L reactor and obtained about 8 L (25 wt%) of casting solution, and then produced membrane products with widths of more than 1000 mm in a membrane casting machine. The surface SEM images present a uniform and flat morphology, which indicate the synthesized AEM materials are free of defects.

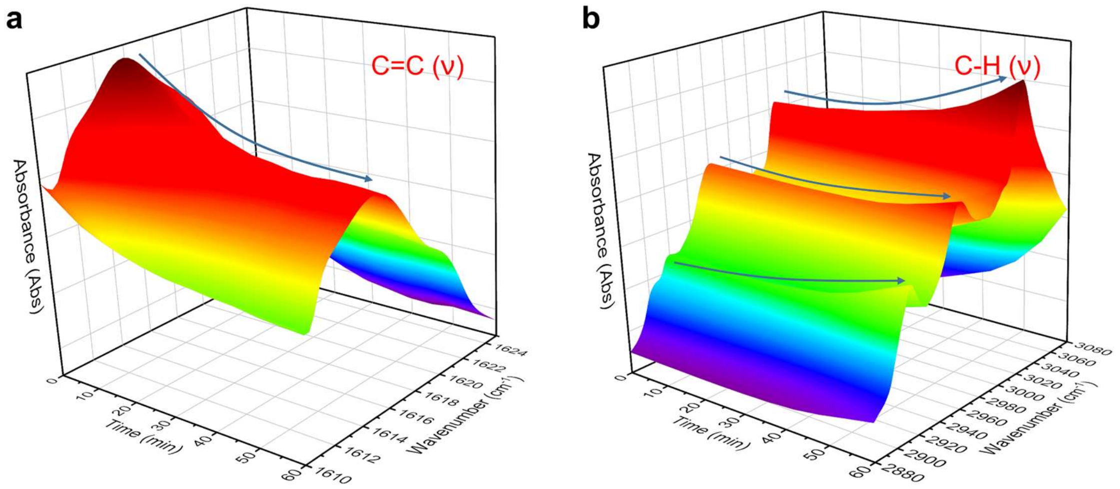

Supplementary Fig. 5 | The thermally-initiated in-situ cross-linking. The time-dependent in-situ FT-IR spectroscopy of MTCP-50 was recorded with heating to 80 ºC at a heating rate of 5 °C min^{-1}.

Notes: The situ FT-IR verified the in-situ cross-linking process of terminal vinyl groups under thermal initiation. The in-situ FT-IR spectroscopy suggests that the cross-linking reaction occurred gradually with the temperature increasing from 30 ºC to 80 ºC. The intensity of the C=C stretching vibration at 1617 cm^{-1} shows a trend of decreasing first and then flattening with increasing temperature. Correspondingly, the overlapping C-H vibrational intensities between 2880 and 3080 cm^{-1} are increased due to methylene and methine groups generated in the thermal cross-linking reaction.

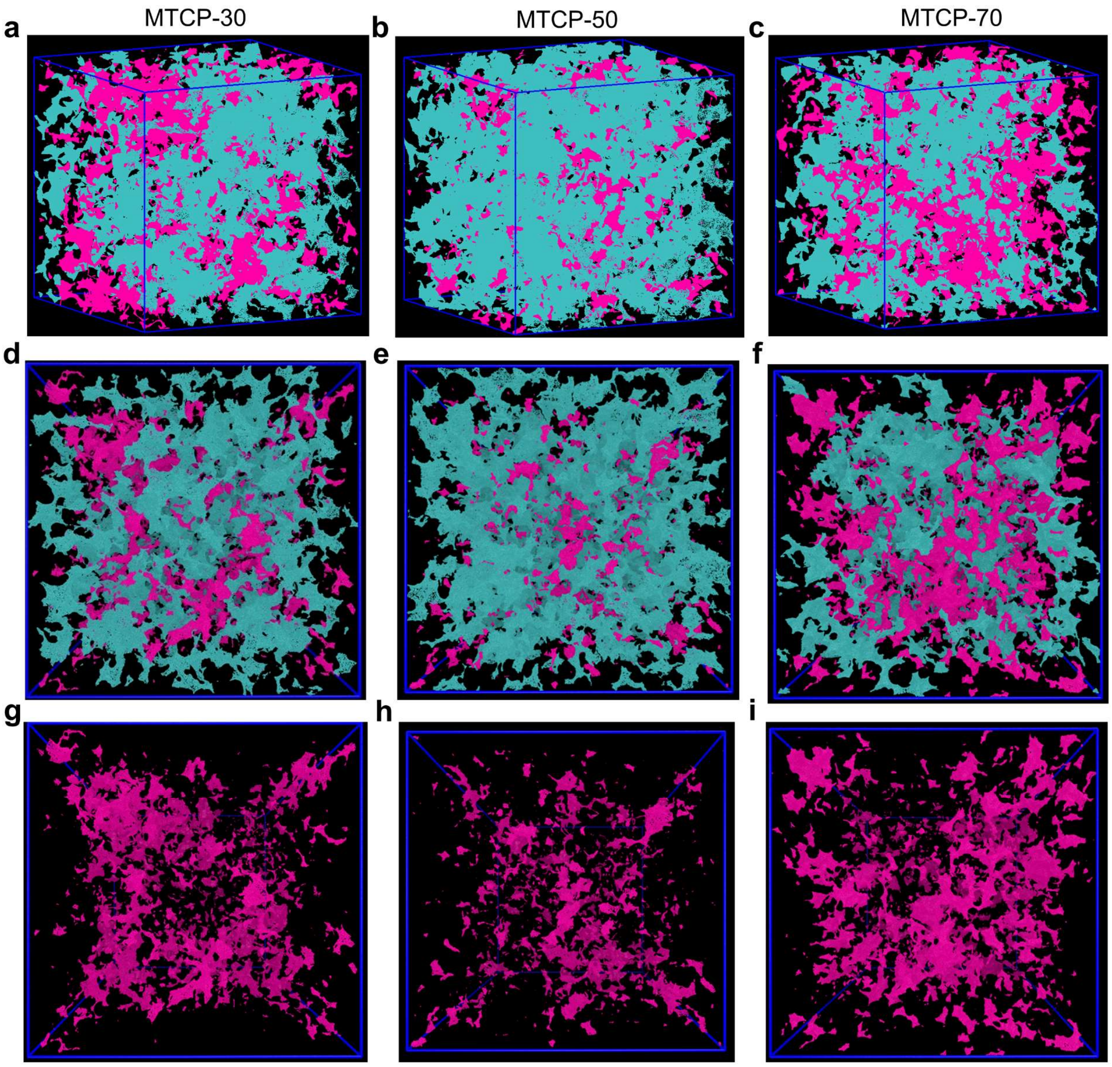

Supplementary Fig. 6 | Structural analysis of amorphous polymer models. a-c, Three-dimensional views of three amorphous cells. Cell size: 100 × 100 × 100 Å. **d-f,** Accessible (teal) and non-accessible (magenta) surface area for MTCP-x series using a 2.0 Å probe diameter. **g-i,** Non-accessible (magenta) surface area for all polymers in the MTCP-x series using a 2.0 Å probe diameter.

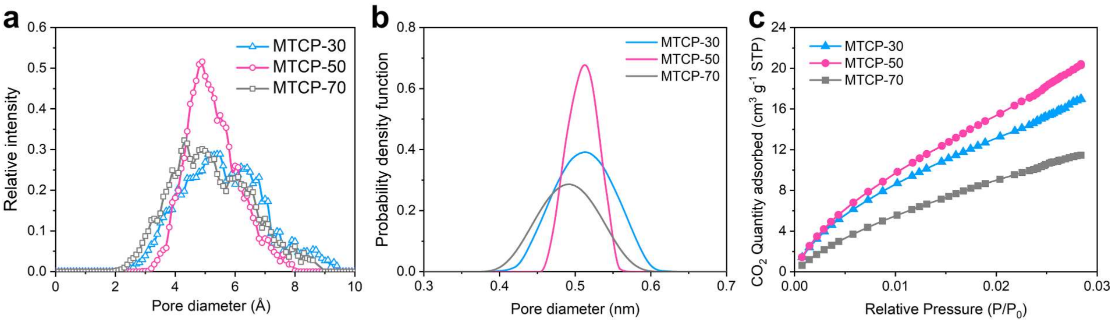

Supplementary Fig. 7 | a, Calculated pore size distribution of the MTCP-x series. **b,** Pore distributions obtained via CONTIN analysis from PALS. **c,** The CO_2 adsorption isotherms of MTCP-x AEM at 298.15 K.

Supplementary Table 1. Characterization of the amorphous polymer models for the three MTCP-x polymer AEMs. The diameters of the largest included sphere (DI), largest free sphere (DF), and largest included sphere along the free sphere path (DIF) are reported, as well as the surface area (SA) with diameter probes of 2 Å.

	**MTCP-30**	**MTCP-50**	**MTCP-70**
**Initial packing density (g cm^3)**	0.4	0.4	0.4
**% of cross-link**	18.6	18.1	18.1
**No. of bonds formed**	201	195	195
**Final bulk density (g cm^3)**	1.05307	1.05303	1.05226
**No. polymer chains**	108	108	108
**DI (Å)**	9.53138	8.84911	8.878
**DF (Å)**	3.5955	3.53678	3.62681
**DIF (Å)**	8.81329	7.91366	8.72012
**SA (cm^2 g^{-1})**	2578.69	2610.79	2600.25

Notes: We performed molecular simulation to generate realistic structural models and analyzed their properties to investigate the ultramicropore structure of polymer networks[1]. All constructed models adopted 108 chain segments to approach the real case as much as possible. For the porosity analysis, Zeo++ was used to probe the pore size distribution and the interconnectivity between voids given at a certain probe radius of 2 Å. **Supplementary Fig. 6**a-c are the three-dimensional view of a modeled amorphous cell. **Supplementary Fig. 6**d-f and g-i show interconnected (teal) and disconnected (magenta) voids with respect to a probe with a radius of 2 Å. The computational work shows notable differences in polymer voids between MTCP-x. The MTCP-50 shows moderate voids and greater connectivity between voids. This confirms that a well balance between rigid and twisted monomers leads to enhanced microporosity and interconnectivity within the membrane. In contrast, MTCP-70 shows isolated voids, indicating their lower porosity and close packing of polymer chains. MTCP-30 exhibits more porosity and moderate interconnectivity due to the inefficient packing of rigid monomers. Pore size distributions for MTCP-x derived from these simulations (**Supplementary Fig. 7**a) are consistent with positron annihilation lifetime spectroscopy (PALS) results (**Supplementary Fig. 7**b). It suggests the narrow distributions of ultramicropores between 4.5 Å and 5.6 Å for MTCP-50, whereas MTCP-30 and MTCP-70 have an apparently broader distribution between 4 Å and 6.1 Å, and 3.8 Å and 6 Å, respectively. Both of which are detrimental to ion transport and the barrier property. The CO_2 adsorption, which defines the interconnect microporosity accessible by CO_2 with a kinetic diameter of 3.3 Å was performed at 298.15 K to provide experimental evidence for the connectivity of voids. As shown in **Supplementary Fig. 7**c, the MTCP-50 displays the largest adsorbing capacity, suggesting the interconnected micropores within MTCP-50. Theoretical calculations and experimental investigations demonstrate that the presence of interconnected pores

exhibited by the MTCP-50 could reduce the resistance of ion transport, hence delivering a high ionic conductivity.

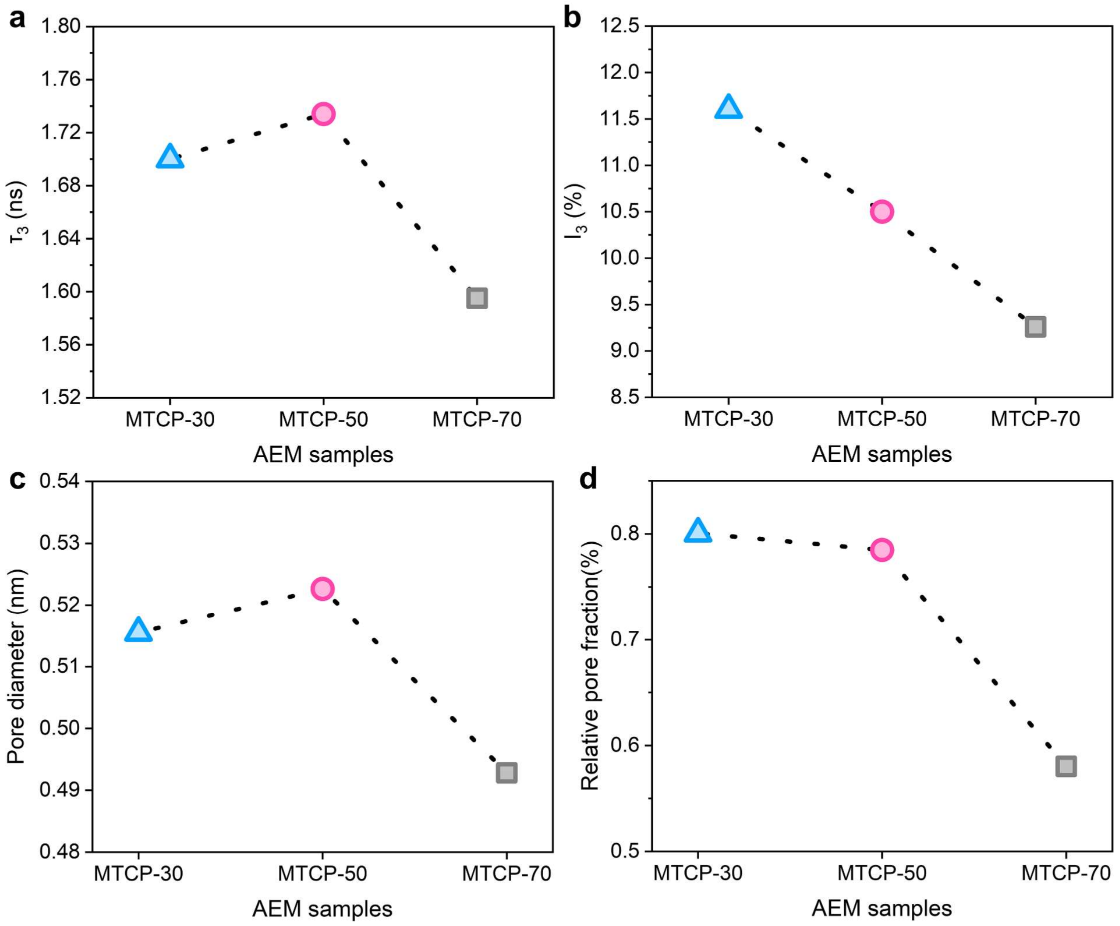

Supplementary Fig. 8 | Positron annihilation lifetime spectroscopy (PALS) of the MTCP-x AEMs. a, Spin-triplet positronium (*o*-Ps) lifetime (τ_3), **b,** *o*-Ps intensities (I_3), **c,** the average free-volume hole diameter, and **d,** the relative free volume fraction of MTCP-x AEMs.

Notes: The positron annihilation lifetime spectrum (PALS) is based on the lifetime measurements of positronium, and refers to the time from the generation of positrons to the annihilation process with electrons located in the free volume of a material[2]. During this process, the average size of the free-volume voids correlates to the spin-triplet *ortho*-positronium (*o*-Ps) third mean lifetime (τ_3). The *o*-PS will preferentially locate within the pore space and then annihilate with electrons on the pore walls of the membrane samples. The pore size determines the time required for the annihilation event to occur. Larger pores result in longer lifetimes. The average spherical pore size from the *o*-Ps lifetime is calculated by Tao–Eldrup equation. Additionally, The lifetime has an associated intensity value (I_3) that corresponds to the relative number of pores within the membrane samples. The relative fractional free volume (FFV_3) is related to the size and number of free-volume holes within the membranes.

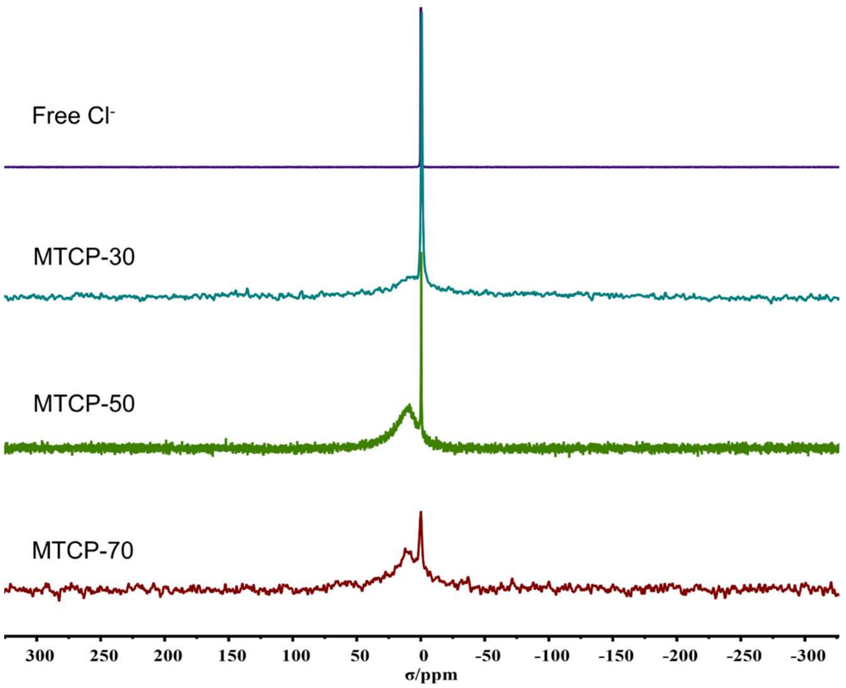

Supplementary Fig. 9 | ^{35}Cl solid state NMR (ss-NMR) measured for MTCP-x.

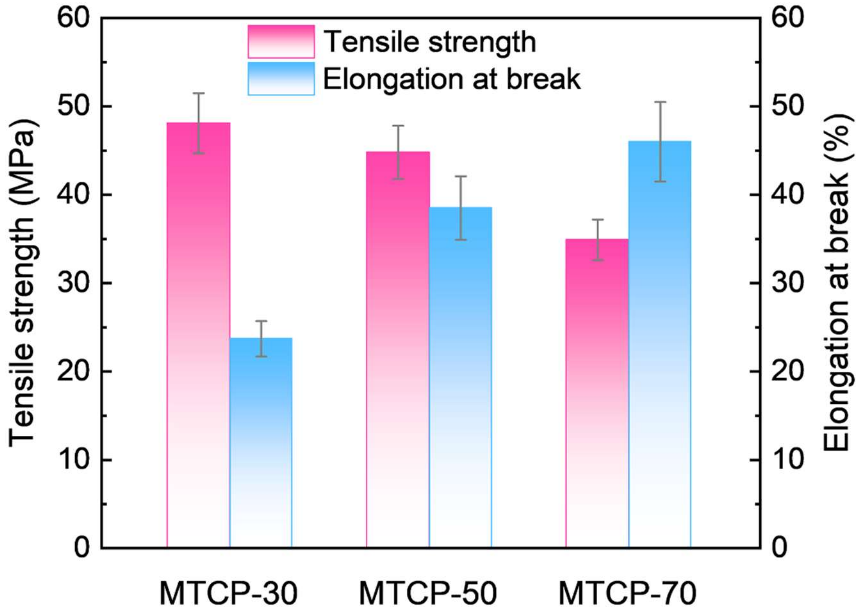

Supplementary Fig. 10 | Mechanical properties of MTCP-x in OH^- form at room temperature (dry state). The error bars represent the standard deviation of mechanical properties.

Notes: Mechanical integrity is one of the most important prerequisites for polymer electrolyte membranes in terms of MEAs fabrication, handling, assembling, and durability. Robust AEMs are required because of the presence of mechanical and swelling stress. Moreover, the AEMs must have a certain elasticity (elongation) to prevent crack formation. Hence, tensile tests were performed to record the tensile strength (Ts) and elongation at break (Eb) of MTCP-x AEMs in OH^- form. As shown in

the stress-strain curves of **Supplementary Fig. 9**, the ultimate Ts of MTCP-30, MTCP-50, and MTCP-70 are 48.1 MPa, 44.8 MPa, 34.9 MPa, and their Eb are 23.7%, 38.5%, 46.0%, respectively. Mechanical deviations of MTCP-x AEMs in terms of strength and toughness are attributed to differences in chain segment rigidity and flexibility resulting from ratio adjustment between p-terphenyl and m-terphenyl. Altogether, the high tensile strength (44.8 MPa) and elongation at break (38.5%) of MTCP-50 can well fulfill the practical application requirement.

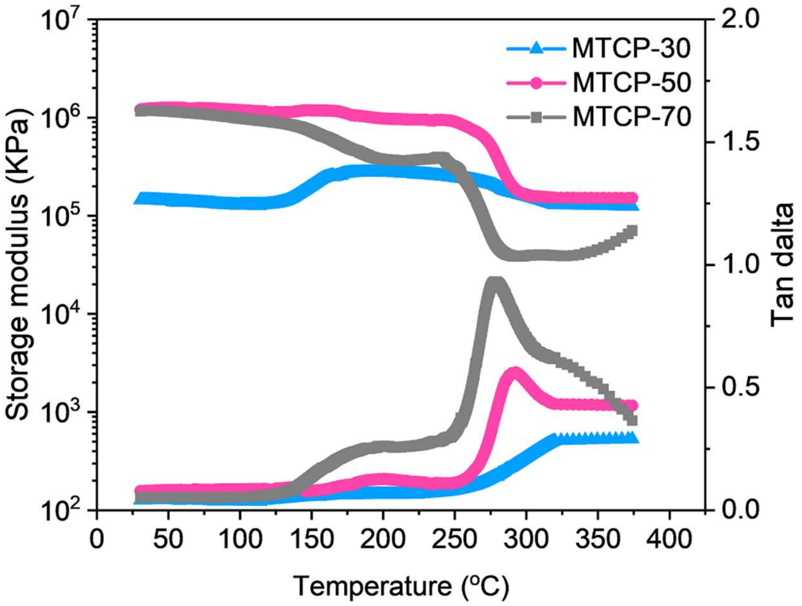

Supplementary Fig. 11 | Storage modulus and Tan delta of MTCP-x membranes.

Notes: The dynamic mechanical analysis (DMA) was further conducted to investigate the rheological properties of the MTCP-x AEMs. The high glass transition temperature (Tg) (> 275 °C) indicates the good high-temperature tolerance property of MTCP-x AEMs. The MTCP-50 AEM displays an outstanding storage modulus (E') than other AEMs, even at 250 °C, the E' over 1.0 GPa, which should indicate good processability as well as an excellent thermal properties.

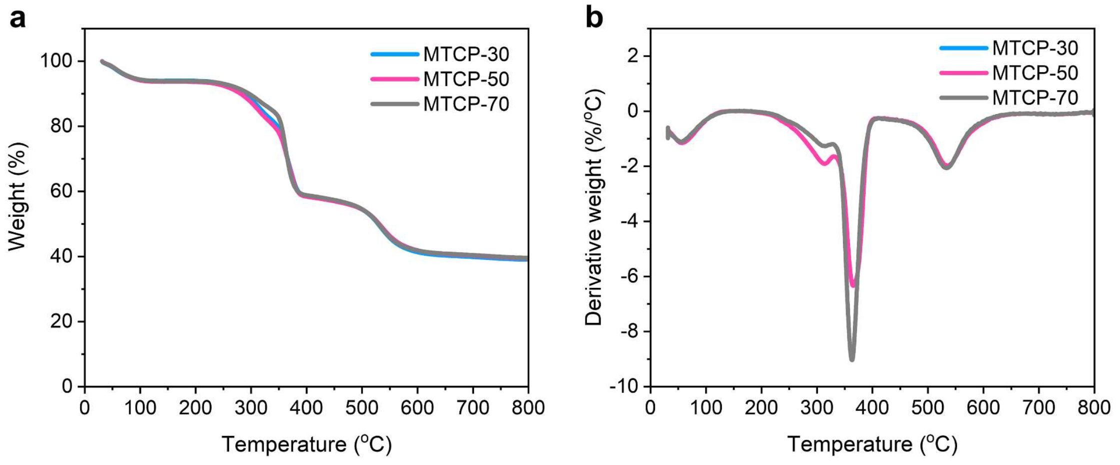

Supplementary Fig. 12 | TGA curves of MTCP-x membranes.

Notes: Thermogravimetric analysis (TGA) revealed the thermal stability of the MTCP-x AEMs. In the **Supplementary Fig. 12**, the first weight-loss stage between 30 to 110 °C is assigned to dehydration, the second stage between 205 to 352 °C is the decomposition of the amino-functional group, and the further weight loss over 391 °C is the decomposition backbone. The thermal degradation data shows that the membrane has a high employ temperature.

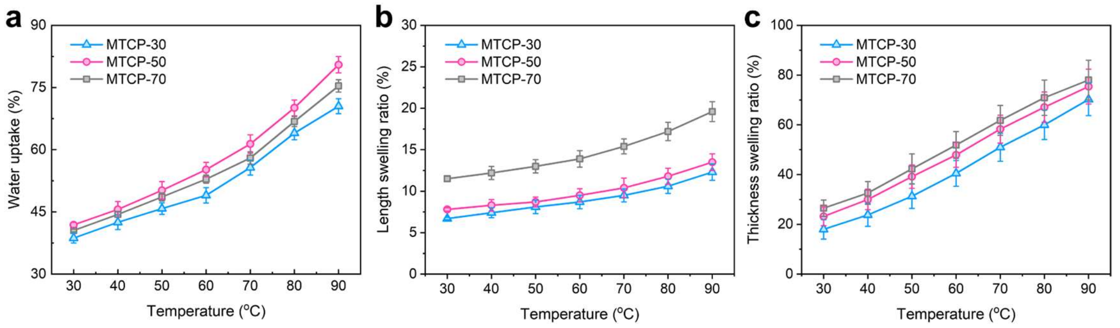

Supplementary Fig. 13 | Temperature-dependent properties of MTCP-x AEMs in water. a, Water uptake, **b**, Length, and **c,** Thickness swelling versus temperature. The error bars represent the standard deviation of water uptake and swelling ratio.

Notes: Typically, water, as an ion transport carrier, affects ion transport efficiency. For fabricated AEMs with similar IEC values, the MTCP-x also displays different water adsorption behavior. For example, at the temperature of 30 °C, the MTCP-50 exhibits large water uptake (WU, 41.8%) but a moderate swelling ratio (SR, 7.8%). The MTCP-70 exhibits moderate WU (40.5%) and large SR (11.5%), while the MTCP-30 shows lower WU (38.7%) and SR (6.7%). This difference in water absorption behavior is governed by the synergistic effects of porosity and hydrophilicity, and high uptake is achieved with polymers that are both microporous and hydrophilic. Undoubtedly, pore filling and expansion by water are expected to generate continuous water/ion conduction channels[3]. Hence, MTCP-50 with moderate water absorption and greater

interconnected water channels (including ionic aggregates water channels and microporous water channels) is more conducive to ion transport. We then studied the swelling behavior of the MTCP-x. For our solution-cast membranes, the length and width usually show similar behavior. Therefore, the 4 × 4 cm membrane samples were cut to measure the swelling ratio (SR) in the length and thickness direction. In the temperature range of 30 to 90 °C, the length swelling of MTCP-50 increased from 7.8 to 13.5%. While the length swelling of MTCP-30 and MTCP-70 increased from 6.7 to 12.3% and 11.5 to 19.6%, respectively. Although length swelling increases almost linearly with WU, it shows a low level. Considering the anisotropic behavior of the membranes, we then analyzed the thickness swelling. As expected, all membranes display a greater thickness swelling. For MTCP-50, the thickness swelling of MTCP-50 increased from 23.2 to 75.4%, while the thickness swelling of MTCP-30 and MTCP-70 increased from 18.0 to 70.2% and 26.5 to 78.0%, respectively. Overall, the MTCP-30 exhibits excellent anti-swelling behavior. The MTCP-30 with more rigid p-Terphenyl tends to be more hydrophobic, coupled with the higher relative free volume fraction and less water absorption resulting in the lower SR. For MTCP-70, it displays a larger SR, because the smaller pore is insufficient to withstand the absorbed water. And MTCP-50 shows moderate anti-swelling behavior due to the more interconnected pores. Additionally, the water absorption behavior of all the membranes shows an increasing trend with temperature. It's worth noting that the MTCP-50 still exhibits good dimensional stability even at high temperatures, which is essential for use in high-temperature.

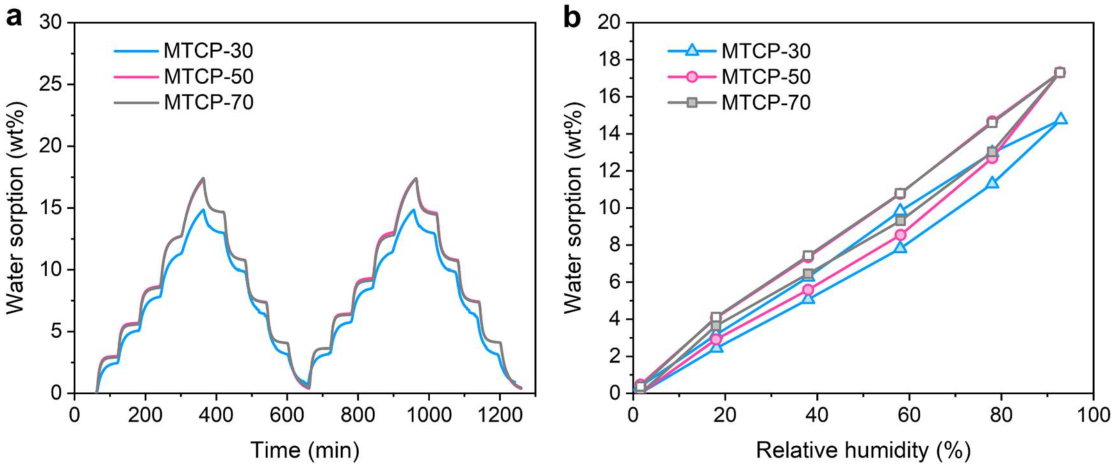

Supplementary Fig. 14 | Dynamic vapor sorption of MTCP-x AEMs. a, Dynamic vapor sorption profiles of MTCP-30, MTCP-50, MTCP-70 AEMs. **b**, Dynamic vapor sorption isotherms of MTCP-30, MTCP-50, MTCP-70 AEMs.

Notes: WU represents the water absorption capacity, but it cannot mean the water transport behavior[4]. Therefore, the water diffusivity of MTCP-x at different RHs was automatically estimated by dynamic water vapor sorption (DVS). As shown in **Supplementary Fig. 14**, the water vapor sorption is significantly lower than their liquid WU (in equilibrium) at different RHs. The hysteresis loop in the absorption and

dehydration curve reflects the characteristics of the microporous polymer[3]. MTCP-30 absorbs less water in comparison to MTCP-50 and MTCP-70, despite the slightly higher micropore volume of MTCP-30. We attribute this low water vapor uptake to the hydrophobicity of MTCP-30[3].

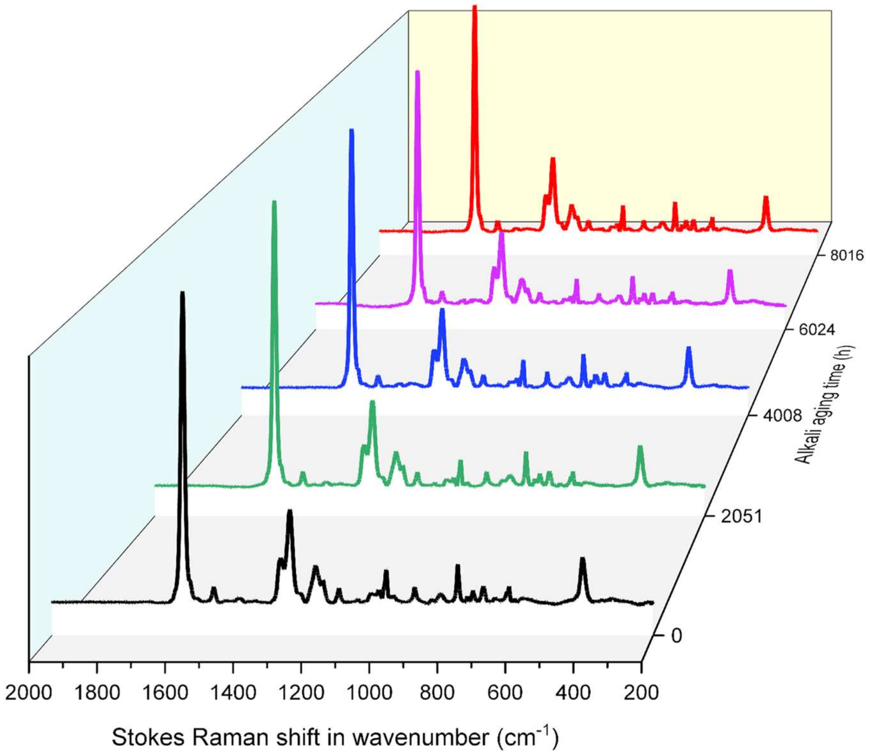

Supplementary Fig. 15 | The Raman spectra of the MTCP-50 AEM after aging in 1 M NaOH at 80 °C at different times. All spectra were normalized to the intensity of the 1605 cm^{-1} peak.

Notes: We performed Raman spectroscopy to analyze the chemical degradation of the MTCP-50 AEM on alkali exposure. The **Supplementary Fig. 15** suggests that the attenuation of signal peak intensity and the appearance of new peak doesn't occur in either the aromatic region or the ester region, which further reveals the excellent stability of MTCP-50 AEM.

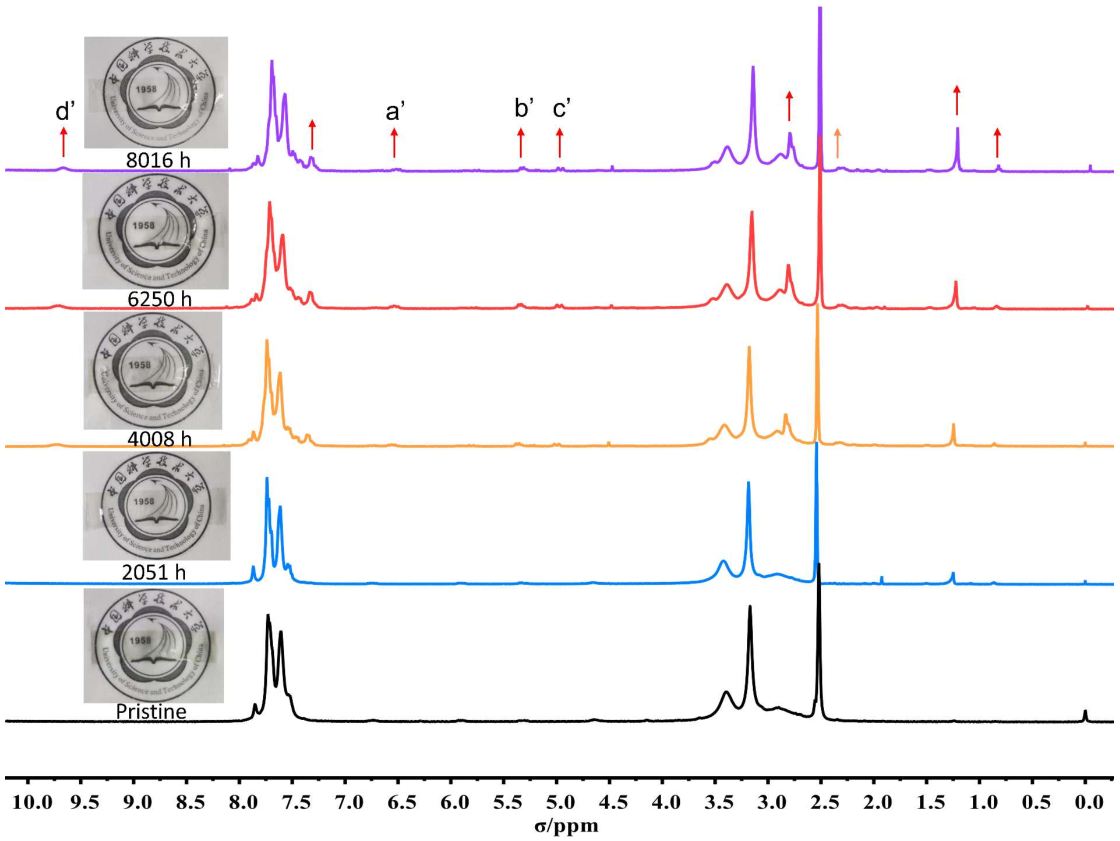

Supplementary Fig. 16 | ^{1}H NMR spectrum and picture of MTCP-50 membrane after alkaline treatment in 1 M NaOH at 80 °C longer than 8000 h.

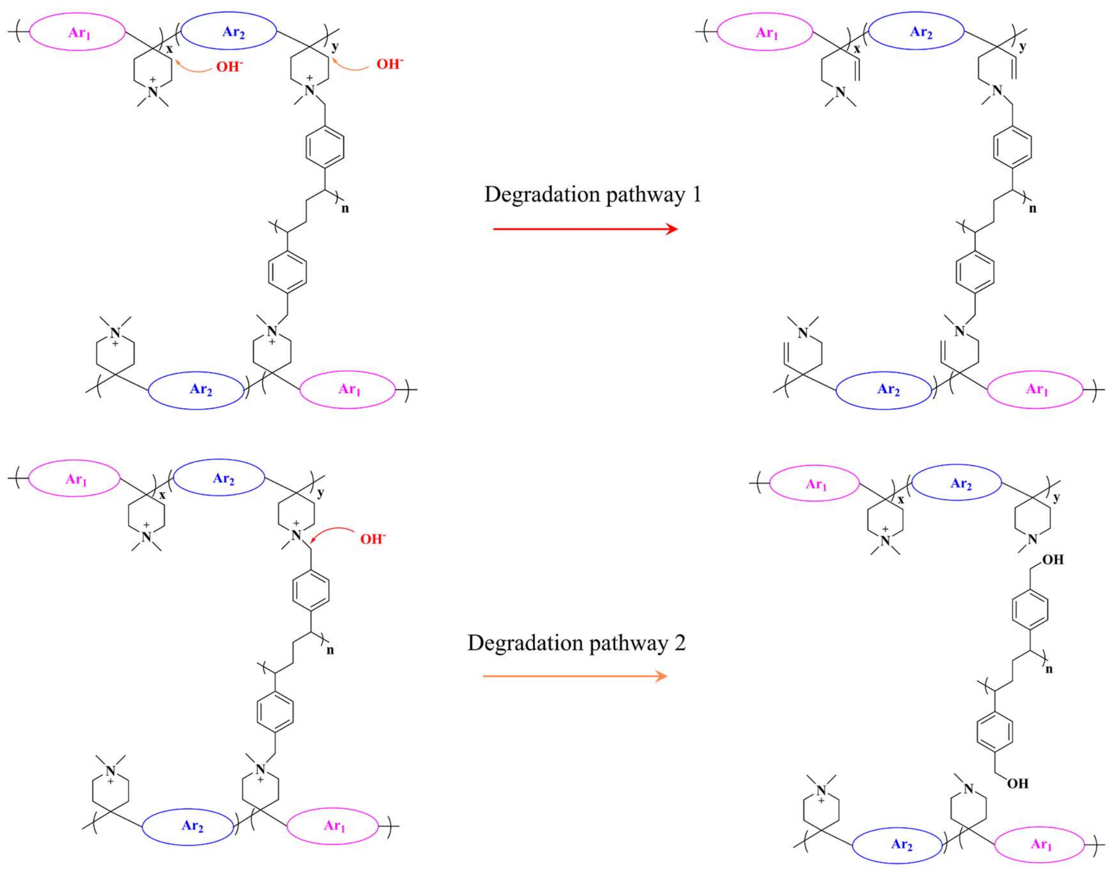

Supplementary Fig. 17 | Possible degradation pathways of MTCP-50: degradation pathway 1 is ring-opening and degradation pathway 2 is S_N2.

Notes: The chemical stability of AEM, mainly in terms of alkaline stability, is the key to the long-lifespan operation of AEM in electrochemical devices. Major strides have been made in improving AEM stability. Despite progress, significant longevity challenges remain for the stability of hydroxide-conducting membranes, especially at higher temperatures. According to the results of high-throughput testing of current existing AEMs by the National Renewable Energy Laboratory (NREL)[5], most AEMs show severe deformation and even fracture along with color changes (in some cases) after alkali aging for 1000 h at 80 ºC in 1 M KOH. To enable robust operation over thousands of hours in high temperatures and alkaline environments, AEMs with polymeric backbone without degradable heteroatoms and a stable cation are urgently needed. For our produced MTCP-50 AEM, an acceleration test over 8000 h was performed by immersing the AEM under high pH conditions (1 M NaOH solution) and high temperature (80 ºC). The OH^- conductivity was measured before and after the durability experiment to quantify the degree of degradation. Additionally, we analyzed the degradation of the MTCP-50 AEM at different alkali aging stages. No visible changes were observed from the photos taken at different periods. All the membranes maintain transparency and are free of defects. ^1H NMR was performed to identify the chemical structure integrity. As shown in **Supplementary Fig. 17**, new signals that emerged at 4.9, 5.3, 6.5 and 9.7 ppm might ascribe to the suffering from ring-opening E2 reaction (the degradation pathway 1)[6, 7]. Additional new signals appeared at 0.8, 1.2,

2.8 and 7.3 ppm, corresponding to the structural rearrangements caused by the loss of charged centers[6]. New signal emerged at 2.08 ppm corresponding to S_N2 degradation [7](the degradation pathway 2). Hence, the possible degradation paths are summarized in **Supplementary Fig. 17** according to the signal peaks observed in ^{1}H NMR spectrum. Notably, only small degradation signals are detected on the ^{1}H NMR spectrum, indicating the excellent alkaline stability of MTCP-50, which is also matched with the OH^- conductivity test results.

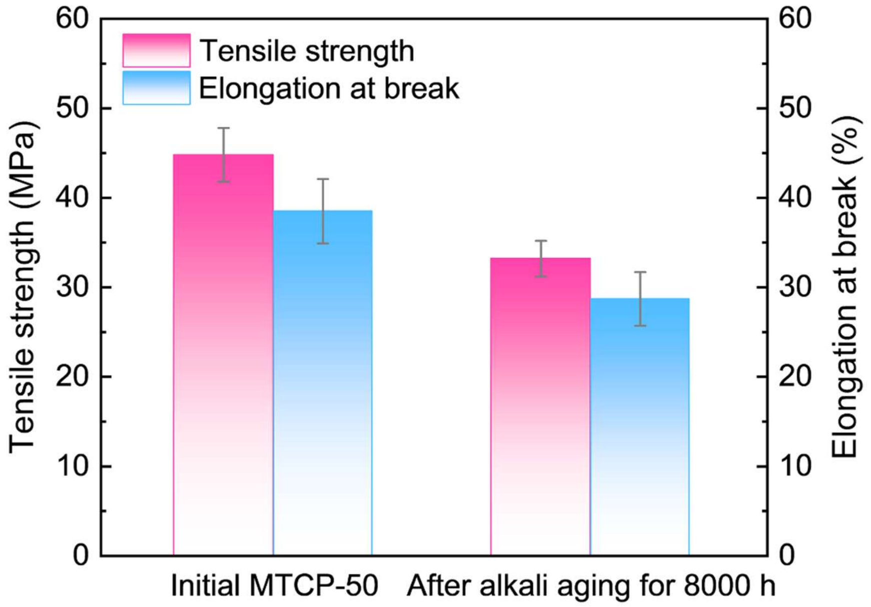

Supplementary Fig. 18 | The variation of mechanical properties (in OH^- form, room temperature and dry state) of MTCP-50 in 1 M NaOH at 80 ºC over 8000 h. The error bars represent the standard deviation of mechanical properties.

Notes: Further mechanical testing is required to expand upon the alkali aging of the membranes. As shown in **Supplementary Fig. 18**, the Ts and Eb are 30.5 MPa and 32.2%, respectively, of MTCP-50 after alkali aging in 1 M NaOH, 80 ºC over 8000 h. The MTCP-50 maintains excellent mechanical strength of ~80% (OH^- form) after alkaline stability testing over 8000 h, which suggests that the present AEM is sufficiently mechanically tough for AEMWEs/AEMFCs applications. The ~20% mechanical loss may be attributed to the fracture of benzyl carbon (the degradation pathway 2) which destroys the cross-linking structure of the MTCP-50.

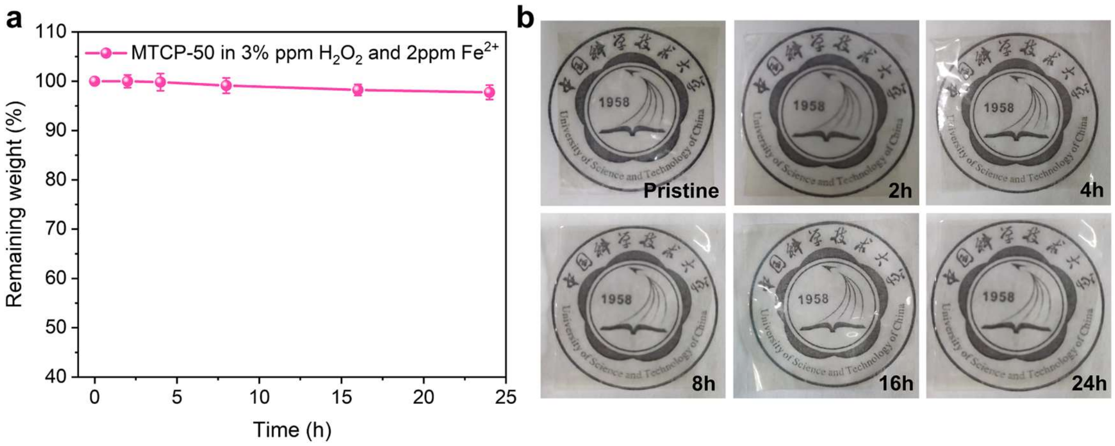

Supplementary Fig. 19 | a, Remaining weight of the MTCP-50 AEM sample after Fenton's test. The AEM sample was immersed and sealed in an aqueous solution of 3% H_2O_2 and 2 ppm Fe^{2+} under 80 ºC. **b,** Photographs of MTCP-50 AEM at different time intervals after Fenton's test. The error bars represent the standard deviation of membrane remaining weight.

Notes: The MTCP-50 AEM sample was immersed in Fenton's reagents for up to 24 h with high weight retention of 97.74%, which is comparable to Nafion. In addition, the MTCP-50 samples keep transparent and maintain mechanical integrity. These results confirmed the excellent oxidation stability of MTCP-50 AEM, which enables it to resist degradation caused by radical species.

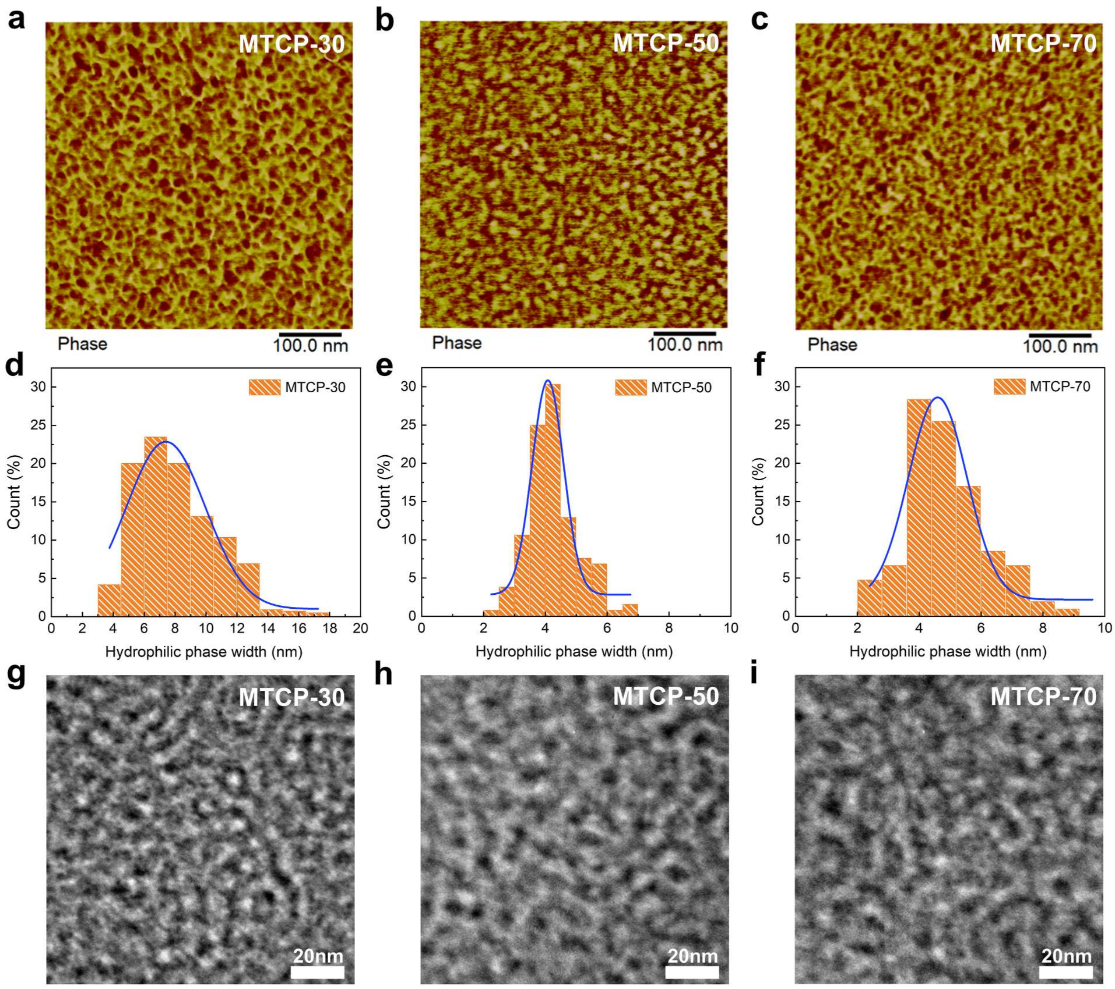

Supplementary Fig. 20 | Microphase morphology of AEMs. **a-c,** AFM of MTCP-x (x=30,50,70). **d-f,** Distribution of hydrophilic phase width of MTCP-x analyzed by the Nano Measurer. **g-i,** TEM of MTCP-x. The bright and dark parts correspond to hydrophobic and hydrophilic regions, respectively.

Notes: The spatial arrangement and length scale of the distribution of ionic and non-ionic segments affect the ion transport efficiency[8]. Hence, the electron microscopy, including AFM and TEM were performed to gain the surface and interior self-assembly morphological features of MTCP-x AEMs. As shown in **Supplementary Fig. 20**, all MTCP-x AEMs exhibit phase separation morphology, wherein, lighter and darker segments correspond to the hydrophobic domains (composed of polymer backbone) and hydrophilic ionic domains (consisting of ionic groups), respectively. The analysis of the distribution of hydrophilic ion channels measured by Nano Measurer suggests more homogeneous ion channels within MTCP-50 AEM, accelerating the ion transport efficiency.

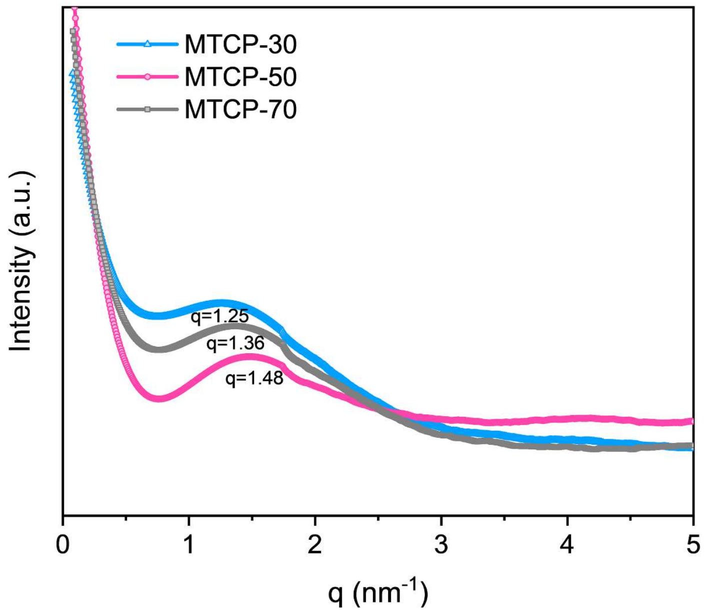

Supplementary Fig. 21 | Small-angle X-ray scattering (SAXS) patterns of MTCP-x AEMs.

Notes: Scattering patterns were further conducted to qualitatively determine the size of the hydrophilic channels. For MTCP-x AEMs, a broad scattering feature is observed. In MTCP-50, a peak maximum is at q = 1.48 $Å^{-1}$. Using d = 2π/q, we could get a mean distance of ca. 4.24 nm. For the sample of MTCP-30 and MTCP-70, the broad peak appears at q = 1.25 $Å^{-1}$ and 1.36 $Å^{-1}$, indicating the mean distance of the sample is around 5.02 nm and 4.62 nm, respectively. In the meantime, peaks show a significant enhancement of intensity, particularly for MTCP-50, which indicates the presence of more continuous hydrophilic channels.

Quantum chemical studies of redox-active molecules were performed using density functional theory (DFT) implemented in GAUSSIAN 16 package1[9]. Geometry optimization and frequency analysis were calculated at B3LYP hybrid functional2 with 6-31G(d) basis sets.

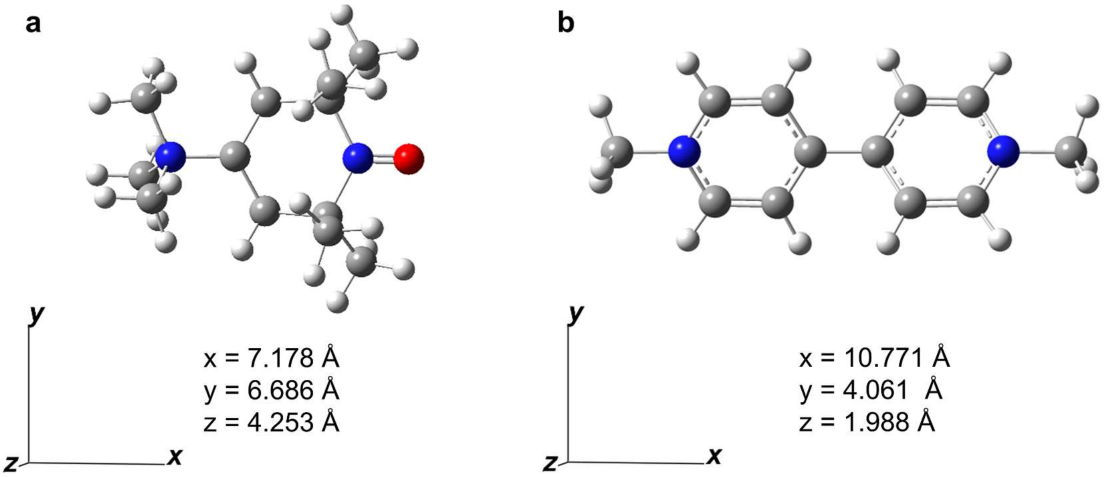

Supplementary Fig. 22 | Optimized molecular structures of redox-active molecules.

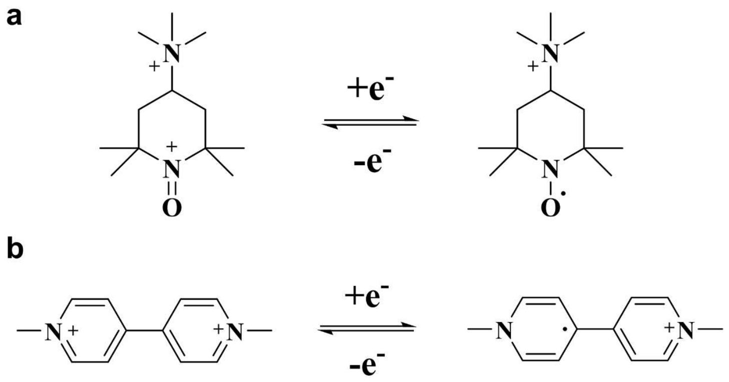

Supplementary Fig. 23 | The electrode reactions of **a,** TEMPTMA (positive electrolyte) and **b,** MV (negative electrolyte).

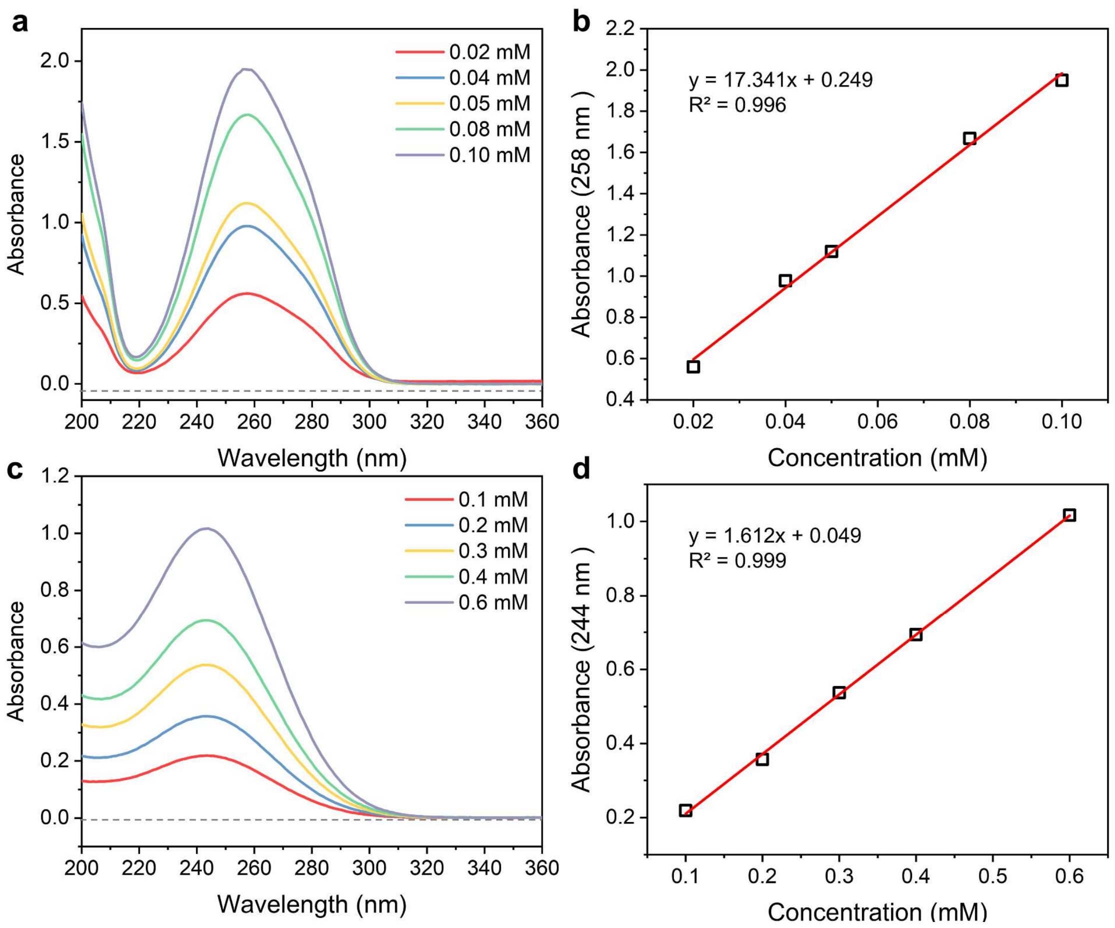

Supplementary Fig. 24 | UV-Vis calibration lines for determination of the concentration of MV solution. a, The UV-Vis absorbance of MV at 257 nm varies with the concentration of aqueous solution. **b,** UV-Vis spectra of MV of different concentrations. **c,** The UV-Vis absorbance of TEMPTMA at 244 nm varies with the concentration of aqueous solution. **d,** UV-Vis spectra of TEMPTMA of different concentrations.

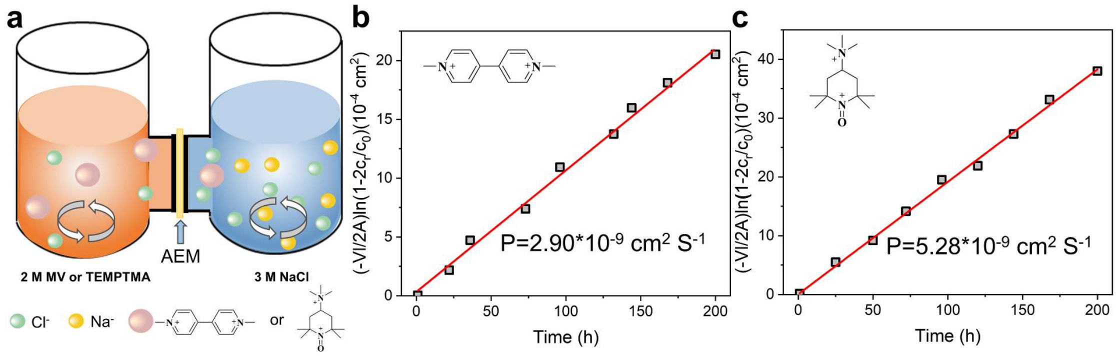

Supplementary Fig. 25 | The permeation of MV TEMPTMA across MTCP-50 membrane using concentration-driven dialysis diffusion H-cells. a, Schematic diagram of the two-compartment diffusion cell. Normalized receiving side concentration (Cr) of **b,** MV and **c,** TEMPTMA as a function of time for MTCP-50 AEM.

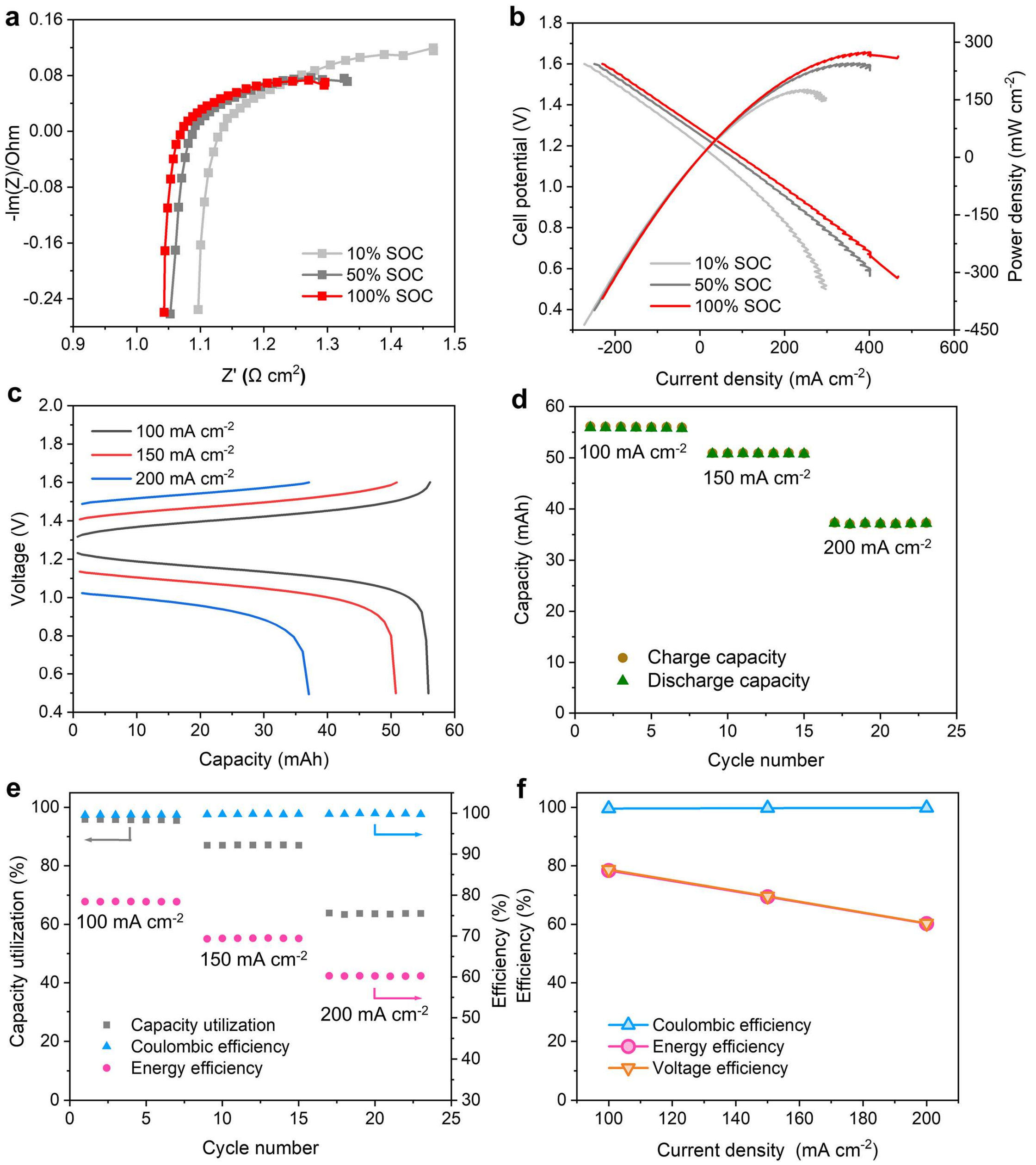

Supplementary Fig. 26 | Performance of the 0.5 M MV/TEMPTMA cell assembled with MTCP-50 AEM. a, EIS spectra and **b,** polarization curves at varied SOC. **c,** Representative charge and discharge curves at current densities from 100 mA cm^{-2} to 200 mA cm^{-2} for the AORFB. **d,** Capacity versus cycling numbers of the AORFB at current densities from 100 mA cm^{-2} to 200 mA cm^{-2}. **e,** The capacity utilization, coulombic efficiency and energy efficiency at varied operational current densities. For each current density, seven repetitions are performed to ensure accuracy. Fluctuations were only observed when we switched the current density. **f,** The coulombic efficiency, energy efficiency, and voltage efficiency at varied operational current densities.

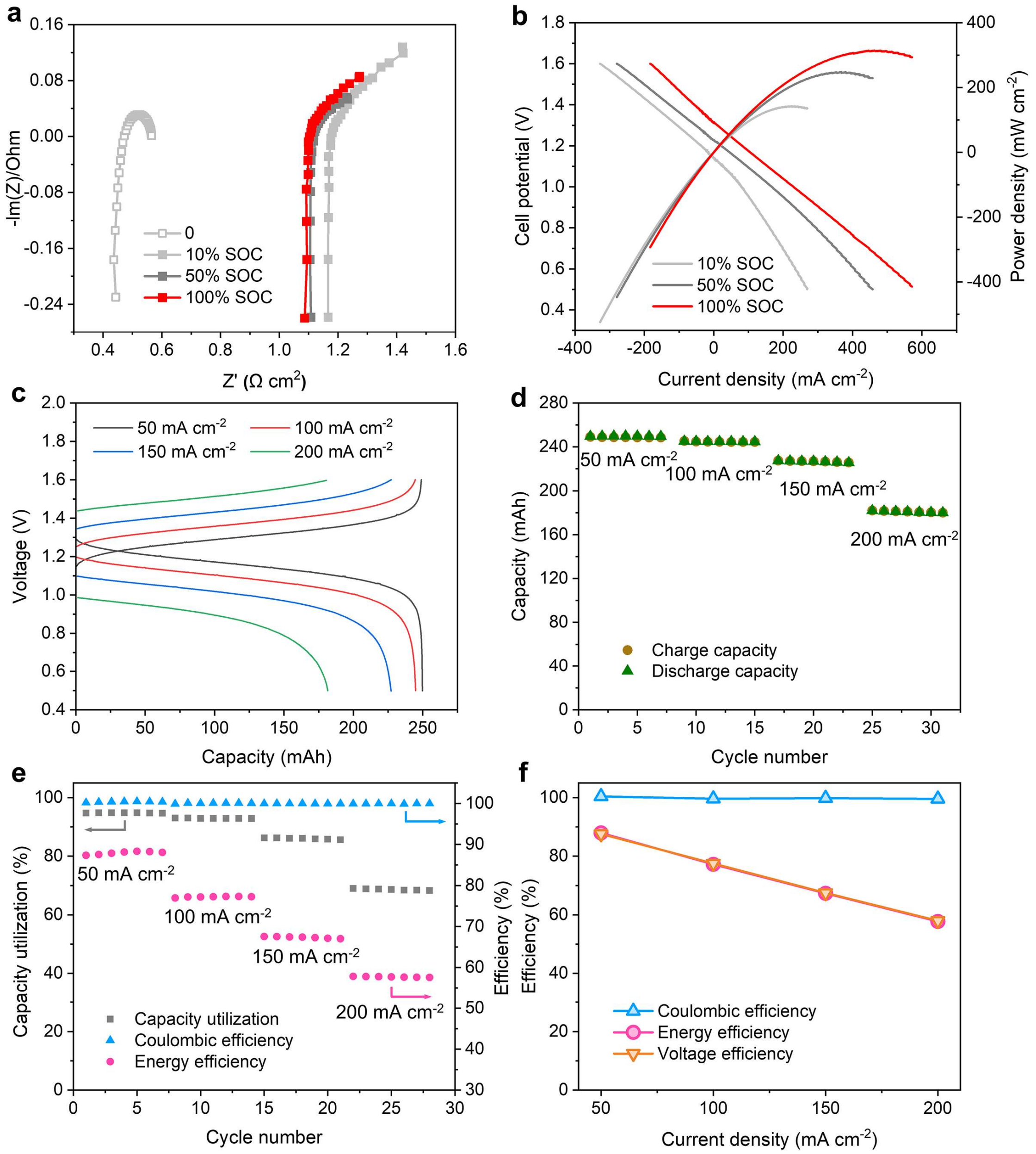

Supplementary Fig. 27 | Performance of the 2.0 M MV/TEMPTMA cell assembled with MTCP-50 AEM. a, EIS spectra and **b,** polarization curves at varied SOC. **c,** Representative charge and discharge curves at current densities from 50 mA cm^{-2} to 200 mA cm^{-2} for the AORFB. **d,** Capacity versus cycling numbers of the AORFB at current densities from 50 mA cm^{-2} to 200 mA cm^{-2}. **e,** The capacity utilization, coulombic efficiency and energy efficiency at varied operational current densities. For each current density, seven repetitions are performed to ensure accuracy. Fluctuations were only observed when we switched the current density. **f,** The coulombic efficiency, energy efficiency, and voltage efficiency at varied operational current densities.

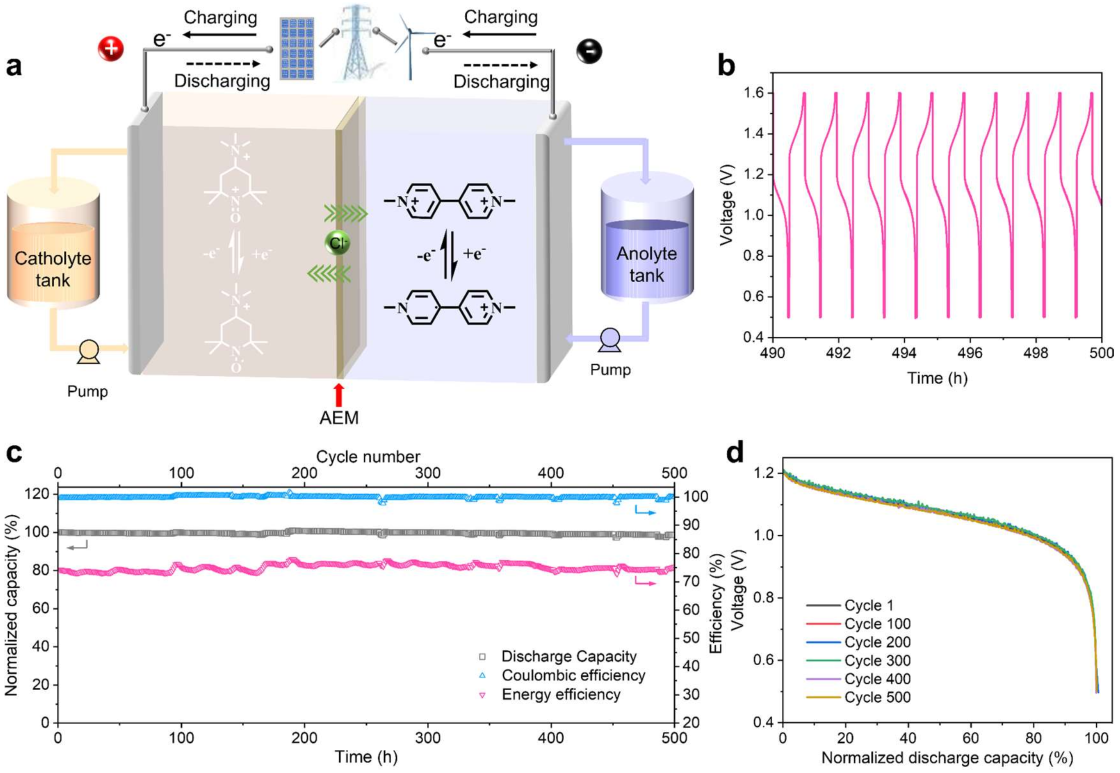

Supplementary Fig. 28 | Long-term stability of the 2.0 M MV/TEMPTMA cell assembled with MTCP-50 AEM. a, Schematic illustration of a MV/TEMPTMA aqueous organic redox flow battery assembled with MTCP-50 AEM for grid-scale energy storage. **b,** Representative cell voltage vs. time curves during the galvanostatic cycling of a MV/TEMPTMA cell assembled with MTCP-50. **c,** Discharging capacity utilization, coulombic efficiency and energy efficiency along 500 h cycles for 2.0 M MV/TEMPTMA cells assembled with MTCP-50 at 100 mA cm^{-2}. (Experiments: Pumped 5 cm^2 test cell; The posolyte comprises 5 mL of 2.0 M TEMPTMA while the negolyte comprises 7.5 mL of 2.0 M MV. The cutoff voltages are 1.6 V and 0.5 V, and a potential hold is applied until the current density falls below 4 $mA \cdot cm^{-2}$) **d,** Normalized discharge capacity of long cycle cells at the 1st, the 100th, the 200th, the 300th, the 400th, and the 500th cycle, respectively.

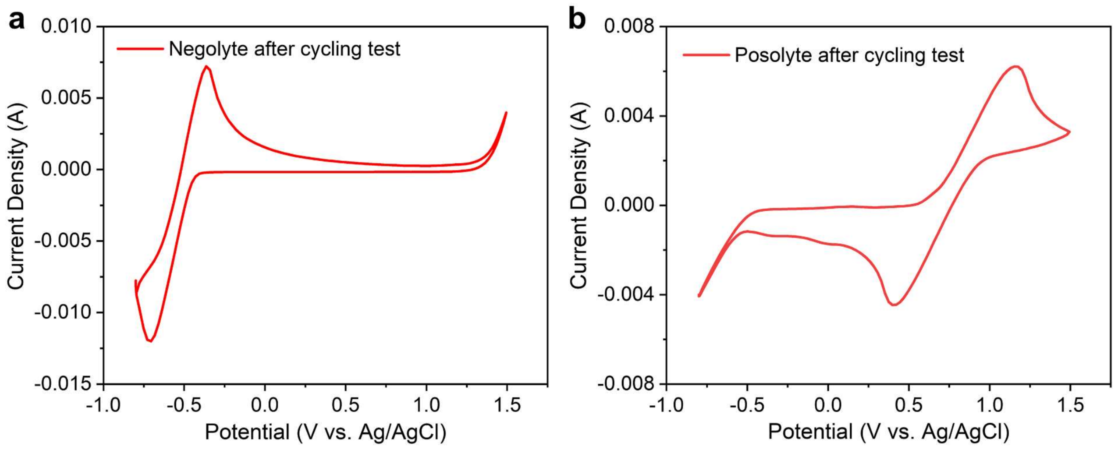

Supplementary Fig. 29 | CV curves of **a,** negolyte and **b,** posolyte after 500 h cycles.

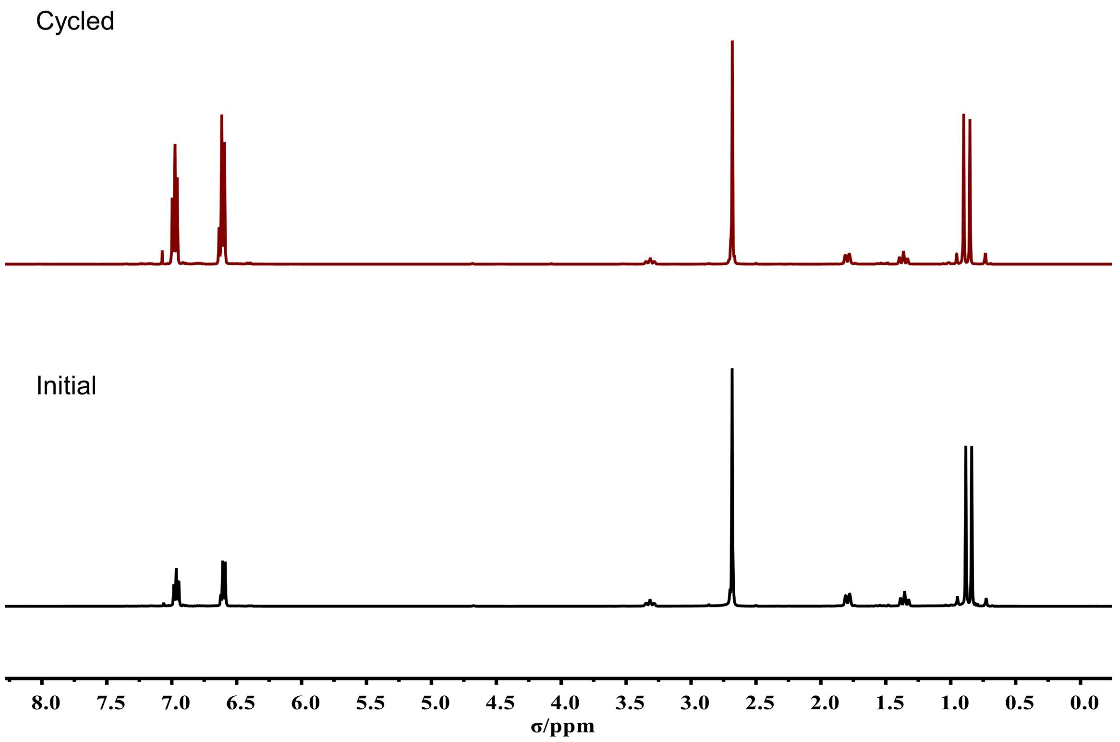

Supplementary Fig. 30 | ^{1}H NMR spectra of initial and after 2.0 M cell cycles of TEMPTMA electrolyte. TEMPTMA was reduced by phenyl hydrazine prior to characterization and the peaks in the region from 6.6 ppm to 7.0 ppm belongs to phenyl hydrazine.

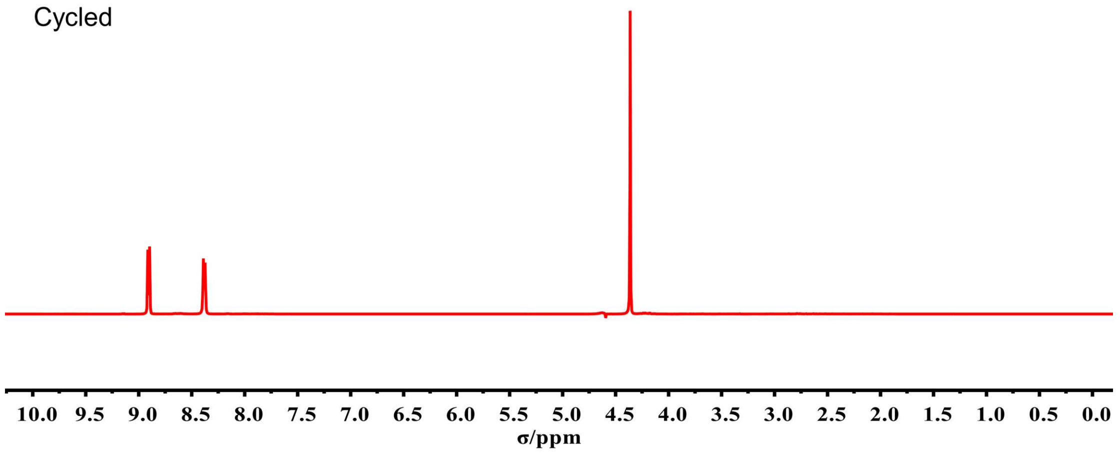

Supplementary Fig. 31 | ^{1}H NMR spectra of MV electrolyte after 2.0 M cell cycles.

Notes: To study the crossover of the redox-active molecules, cyclic voltammograms (CV) and NMR spectra were examined after 500 h cycles at 2.0 M cell. From the CV curves in **Supplementary Fig. 29**, no penetration of positive and negative electrolytes are observed after cycling. Then, for ^{1}H NMR test, 100 uL of the initial and cycled TEMPTMA or MV electrolytes were diluted in d6-DMSO with phenyl hydrazine or d6-DMSO, respectively. As shown in **Supplementary Fig. 30** and **31**, all peaks of cycled electrolyte show no loss of integration area with respect to initial electrolyte. Additionally, there is no new decomposition peak and no penetration peak in cycled electrolytes. All these results indicate the excellent redox-active molecules impermeability of the MTCP-50, which is crucial for the long-term stable operation.

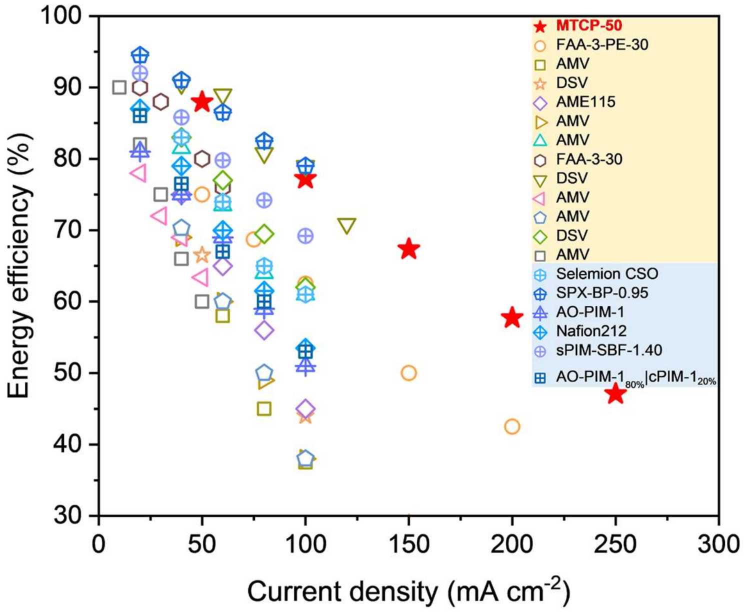

Supplementary Fig. 32 | Summary of energy efficiency versus current density of current state-of-the-art NAORFBs (The yellow and blue backgrounds are AEMs and PEMs, respectively). Refer to Supplementary Table 5.

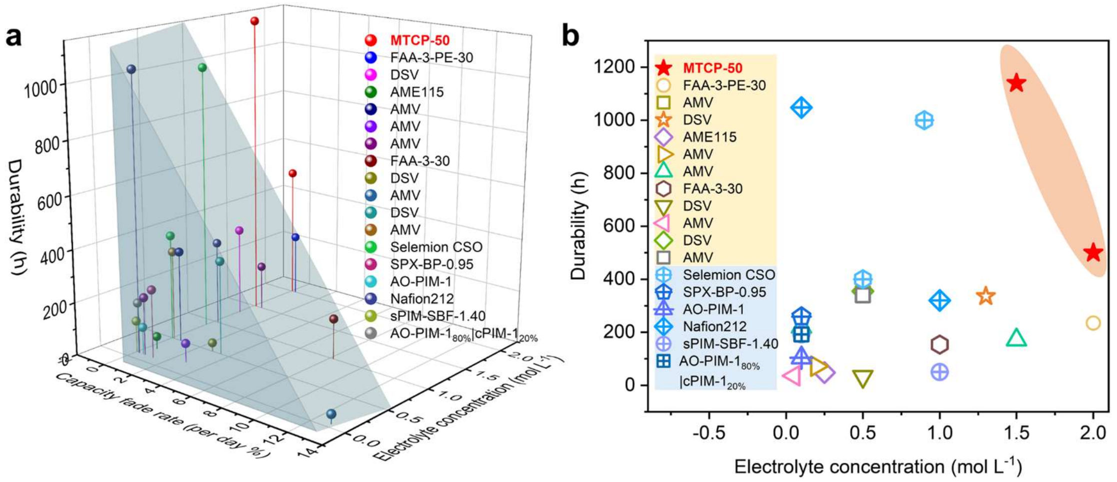

Supplementary Fig. 33 | a, Summary of capacity fade rate-electrolyte concentration-durability of current state-of-the-art NAORFBs (The selemion CSO, SPX-BP-0.95, AO-PIM-1, Nafion 212 are the representative PEM at present). **b,** Summary of long-term durability and electrolyte concentration (The yellow and blue background colors are representative AEMs and PEMs, respectively). Refer to Supplementary Table 5.

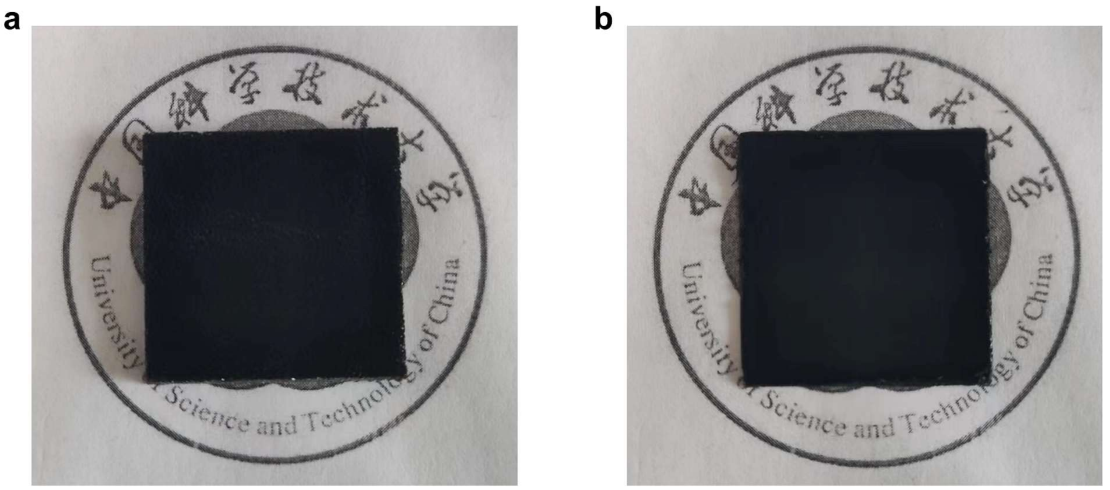

Supplementary Fig. 34 | Photographs of catalyst used for AEMWE. a, NiFe for OER. **b,** Pt/Ru/C for HER.

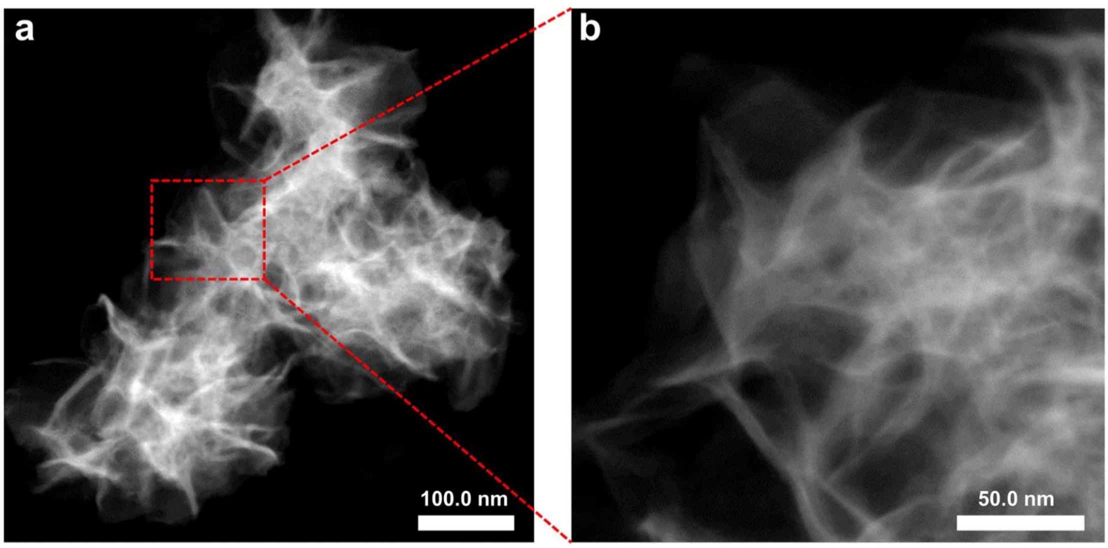

Supplementary Fig. 35 | Dark-field TEM characterizations of OER catalytic.

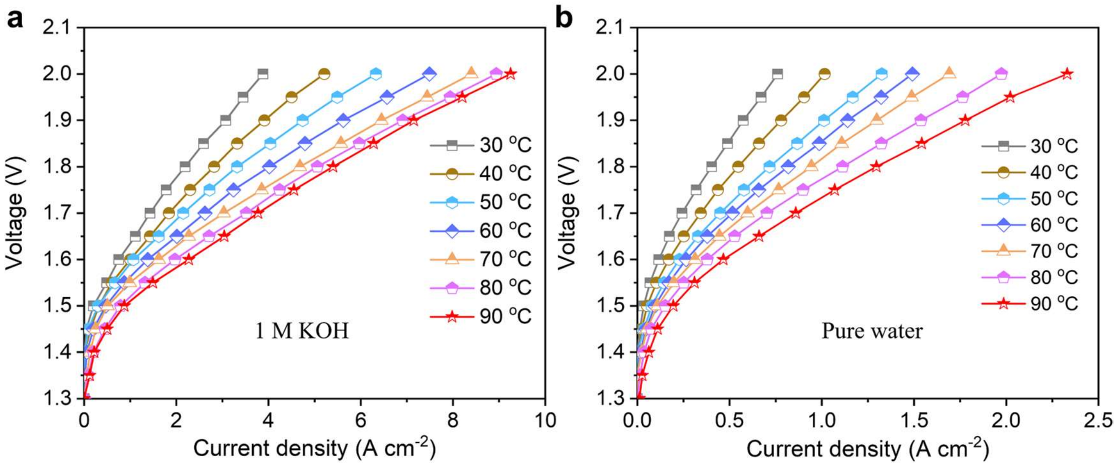

Supplementary Fig. 36 | AEM electrolyser performance versus temperature. a, Performance of MTCP-50 based AEMWE feed with 1 M NaOH. **b,** Performance of MTCP-50 based AEMWE feed with pure water.

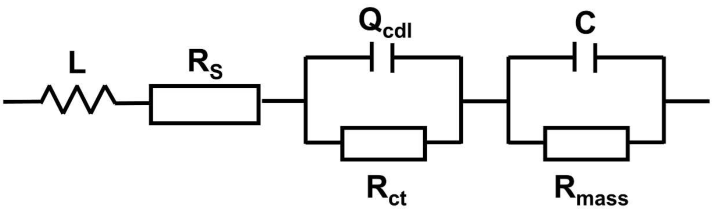

Supplementary Fig. 37 | Schematic diagram of electrical circuits.

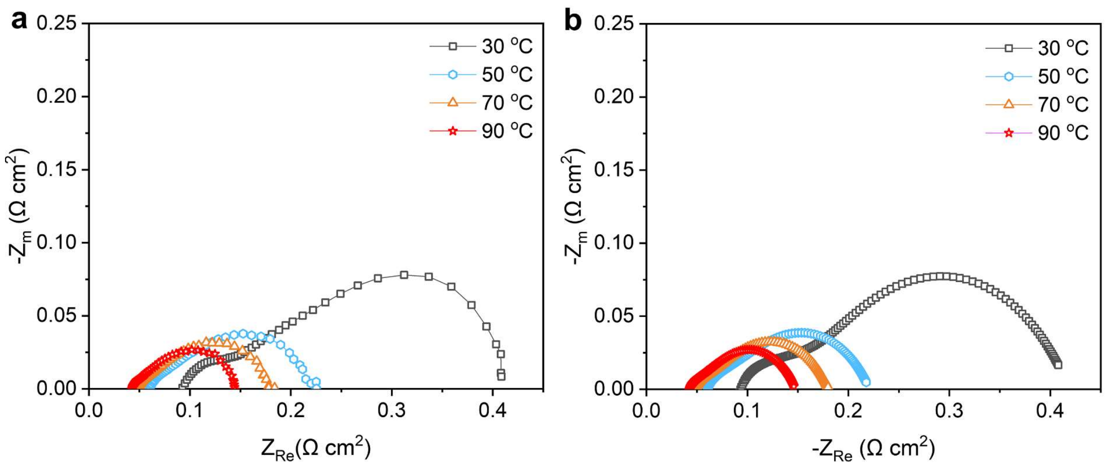

Supplementary Fig. 38 | a, EIS and **b,** EIS fitting spectra of AEMWEs at different temperature and 1.6 V with 1 M KOH feeding.

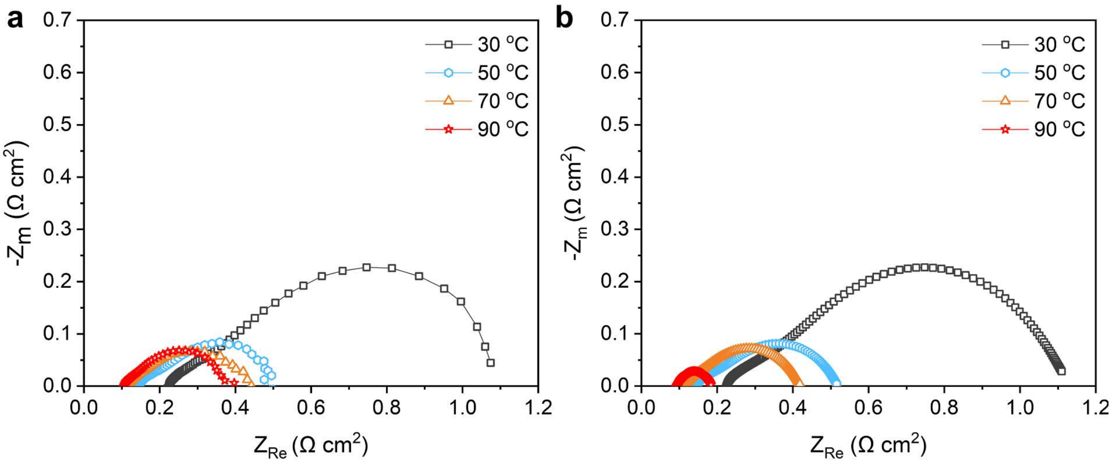

Supplementary Fig. 39 | a, EIS and **b,** EIS fitting spectra of AEMWEs at different temperatures and 1.6 V with pure water feeding.

Notes: The significant improvement in AEMWE performance with increasing temperature can be attributed to the following aspects: 1. The greatly improved ionic conductivity reduces the ohm impedance; 2. The accelerated reaction kinetics of HER and OER benefits by reducing the charge transfer resistance; 3. The rapid desorption of gas from the electrode greatly reduces the mass transfer resistance. We performed Electrochemical Impedance Spectroscopy (EIS) to explain this process. As can be seen from **Supplementary Fig. 38 and 39**, the charge transfer resistance significantly decreases while the ohm resistance also decreases with the increase in temperature.

Supplementary Table 2. The overall error value in EIS fitting process.

	30 °C	50 °C	70 °C	90 °C
1 M NaOH	3.99%	1.77%	2.12%	1.63%
Pure water	1.50%	1.82%	3.99%	1.62%

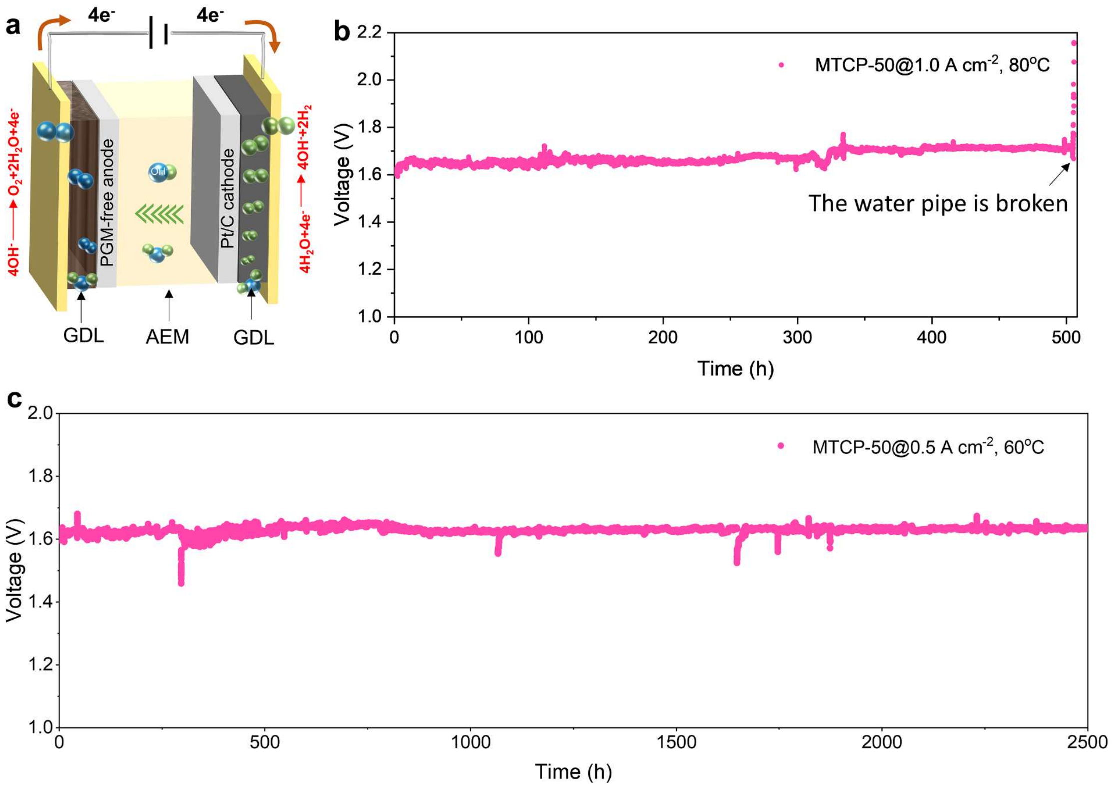

Supplementary Fig. 40 | Long-term stability of the 1.0 M KOH feeding cell assembled with MTCP-50 AEM at different temperature and current density. a, Schematic illustration of water electrolysis for producing H_2 assembled with MTCP-50 AEM. **b,** Long-term durability of AEMWEs under 1.0 A cm^{-2} at 80 °C. **c,** Long-term durability of AEMWEs under 0.5 A cm^{-2} at 60 °C.

Supplementary Table 3. H_2 permeability test report of MTCP-50 AEM with a thickness of 25.8 um.

**Test conditions:** ambient temperature is 31.5 °C, 50 % humidity				**Average value**
**Peak area of standard curve**	422617.93	394235.65	460441.47	425765.017
**Sample peak area**	79736.15	81290.93	80350.78	80459.28
**H_2 volume fraction (ppm)**	46.81934	47.73227	47.18024	47.24395
**H_2 permeability velocity (sccm)**	0.009364	0.009546	0.009436	0.009448
**H_2 permeability (sccm cm^{-2})**	0.0003746	0.0003819	0.0003774	0.0003780
**Standard state**	Temperature: 25 °C	Pressure: 1atm	H_2 density (kg m^{-3})	
			0.082348	

**Experiment state**	Temperature: 25 °C	Pressure: 1atm	0.080592
**H_2 permeability under experiment state (cm^3 cm^{-2} min^{-1})**	**0.00039**		

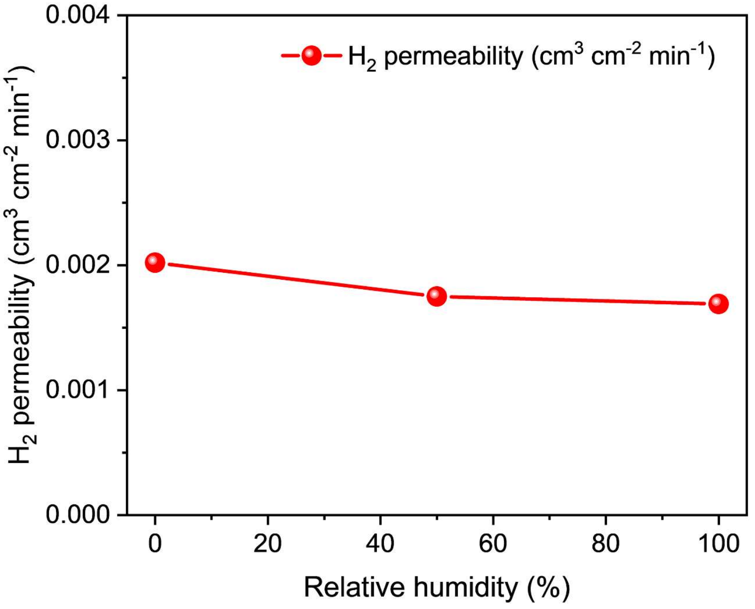

Supplementary Fig. 41 | H_2 permeability of MTCP-50 AEM at 80 °C under different relative humidity.

Notes: For AEMWEs and AEMFCs represented by hydrogen energy, the assembled AEM, in addition to conducting ions, excellent gas impermeability is also required. For AEMWEs, the product crossover will lead to the reduced purity of produced hydrogen, and the subsequent separation process will increase the cost of hydrogen production. For AEMFCs, the reactant crossover will reduce the electromotive force of the electrode, resulting in lower energy output efficiency. When the gas permeates enough and reaches a certain concentration, the heat energy released by direct chemical reaction will lead to continuous degradation of the AEMs, and the danger of explosion will occur in severe cases. Therefore, evaluating the gas permeability of AEMs is critical. Herein, the H_2 permeabilities of MTCP-50 were performed using a Single fuel cell tester connected with a gas chromatograph (GC, Agilent 7890B). As shown in **Supplementary Table 3**, the MTCP-50 shows a lower H_2 permeability of 0.00039 cm^3 cm^{-2} min^{-1} comparable to Nafion (≤0.02 cm^3 cm^{-2} min^{-1}) at 30 °C. Even under 80 °C and different relative humidity, the H_2 permeability of MTCP-50 AEM is still lower than 0.002 cm^3 cm^{-2} min^{-1}. It suggests that the MTCP-50 has an excellent gas barrier property.

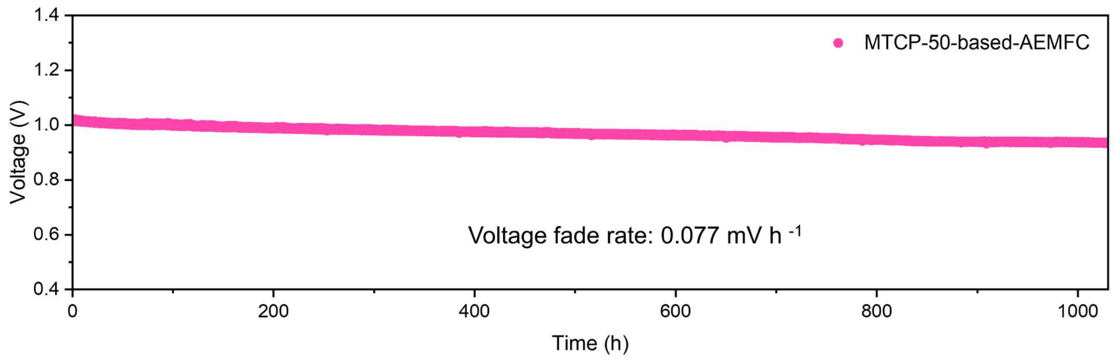

Supplementary Fig. 42 | Open-circuit voltage hold test of MTCP-50-based AEMFC at 60 ºC and 100% RH (H_2/O_2).

Supplementary Table 4. Summary and comparison of the most critical properties of AEMs.

Year	AEM	IEC (mmol g^{-1})	Tensile strength (MPa)	Elongation at break (%)	Swelling ratio (%)		OH^- Conductivity ($mS\ cm^{-1}$)		Alkaline stability time (h)	Conductivity loss ($\times 10^{-2}$ % per h)	Reference
					30 °C	80 °C	30 °C	80 °C			
2015	SEBS-TMA	2.41	14	50	NA		45	102	672	NA	10
2017	m-TPN1	2.15	29	34	10	23	54	112	720	0.129[a]	11
2017	XE-Imd70	2.72	60.2	23.7	NA	62	NA	107	510	6.47[a]	12
2017	LDPE	2.87	29.3	276	22	NA	86	145	672	NA	13
2017	Spiro-ionene 2	4.0	NA		NA		NA	115 (blend with PBI-OO)	1800	NA	14
2018	60-QA-LDH/TCQAPPO	3.69	42	36	21	NA	36	105	500	2.26	15
2018	QPAF-C3	1.24	17	170	NA		35	100	1000	2.7	16

2018	QAPPT	2.49	34.8	39.5	NA		NA	137	5040	0.08	[17]
2019	F20C9N	1.12	NA		26 (25 ^{o}C)	NA	23 (25 ^{o}C)	91	1000	1.7	[18]
2019	PX75-T50	0.91	264	1.8	NA	2.6	51	111.6	720	3.88[a]	[19]
2019	PAP-TP-85	2.37	67	117	7	10	78 (20 ^{o}C)	168	2000	0.15	[20]
2020	QPC-TMA	2.31	NA		9 (25 ^{o}C)	21	77 (25 ^{o}C)	154	1000	Almost no degradation	[8]
2020	GT75-15	3.49	NA		13 (25 ^{o}C)	NA	70 (25 ^{o}C)	153	1020	0.122	[21]
2021	PDTP-25	2.8 (OH^-)	61	32	33.3	48.6	80	165	1536	0.65	[22]
2021	PFTP-13	2.16 (I^-)	48.5 (OH^-)	30.2 (OH^-)	16	119	66	171	2000	0.2	[4]
2021	b-PTP-2.5	2.84	62	35	NA	25.9	89	146.7	1500	0.13	[23]
2022	MM-LPF-OHTP	1.654	22	69	3 (20 ^{o}C)	7.5	83.4	145	4320	Almost	[24]

							(20 ^{o}C)			no degradation	
2023	FPAP-3	2.73	84	40	13.5	15.5	97<br>(40 ^{o}C)	148	2061 (PFAP with 20% fluorinated fluorene monomer)	0.48	25
**2023**	**MTCP-50**	**2.53 (OH^-)**	**44.8 (OH^-)**	**38.5 (OH^-)**	**7.8**	**13.5**	**78.4**	**196.3**<br>**217.0 (90 ^{o}C)**	**8016**	**0.071**	**This work**

[a] Calculated from the capacity fade rate.

Supplementary Table 5. Summary and comparison of representative AORFBs performance and stability.

Year	Membrane	Anolyte / Catholyte	Concentration (mol L^{-1})	Current density (mA cm^{-2})	Energy efficiency (%)	Power density (mW cm^{-2})	No. of cycles (No. of hours)	Capacity fading rate (%)		Reference
								Per cycle	Per day	
2016	FAA-3-PE-30	MV/ TEMPTMA	2.0/2.0	80	67[a]	NA	100 (235[a,b] h)	0.037[a]	0.28[a,b]	26
2017	DSV	BTMAP-Vi/ BTMAP-Fc	1.3/1.3	50	66.5	NA	250 (336 h)	0.0057	0.1	27

2017	AME115	$[(NPr)_2V]Br_4$/ FcNCl	0.25/0.5	60	69	92	100 (48[c] h)	0.01	0.5	28
2017	AMV	MV/FcNCl	0.5/0.5	60	58	100	700（347 h）	0.01	0.62	29
2018	AMV	$[(NPr)_2TTz]Cl_4/N^{Me}$-TEMPO	0.1/0.2	40	69	NA	300 (~72 h）	0.03	2.88	30
2019	AMV	BTMAP-Vi/TMAP-TEMPO	0.1/0.1	60	69	99.03	1000（220 h）	0.007	0.624	31
			0.5/0.5	100	61.8	134	200（80.6 h）	0.01[a]	0.648	
			1.5/1.5	NA	NA	110	250（171.7 h）	0.015	0.552	
2020	FAA-3-30	MV/ 4-OH-TEMPO	0.6/0.6	75	69.7	121.6	250 (67.7[c] h)	0.035	3.1	32
			1.0/3.0	NA	NA	65	70 (153.6[c] h)	0.75	8.2	
2021	DSV	$(APBPy)Cl_4$/ $(TBABPy)Cl_3$	0.5/0.25	80	80.8	NA	100 (33[c] h)	0.04	2.88	33
2021	AMV	BTMAP-Vi/CPL	0.05/0.05	40	69	NA	500 (36 h)	0.04	12.96	34
2022	AMV	$(NPr)_2V/N_2$-TEMPO	1.0/1.0	60	60	114	400 (NA)	0.025	NA	35
2022	DSV	$[PyrPV]Cl_4$/ PyrTEMPO/	0.25/0.5	40	83	317	1000 (355h)	0.05	3.47	36
2022	AMV	C_3-FcNCl/$(NPr)_2VCl_4$	0.5/0.5	50	60	NA	500 (340 h)	0.0037	0.14	37

2019	Selemion CSO	$(NH_4)_4[Fe(CN)_6]$/ $(SPr)_2V$	0.5/0.5	60	74	99.6	500 (400 h)	0.00027	0.01656	38
2020	SPX-BP-0.95	DHAQ/ $K_4[Fe(CN)_6]$	0.1/0.1	100	82.7	243	1000 (259 h)	0.02	1.2	39
2020	AO-PIM-1	2,6-DHAQ/ $K_4Fe(CN)_6$	0.1/0.1	100	51	82.2	400 (104 h)	0.006	0.5	3
2021	Nafion212	SPr-Bpy/ $Na_4[Fe^{II}(Dcbpy)2(CN)_2]$	0.16/0.1	100	54.0	NA	6000 (1050 h)	0.00158	0.217	40
			1.0/1.0	NA			400 (320 h)	0.008	0.25	
2022	sPIM-SBF-1.40	2,6-DPPAQ/ $K_4Fe(CN)_6$	0.1/0.1	100	69	142	2100 (120 h)	0.0000795	0.0335	41
			1.0/0.4		79	303	140 (50 h)0.1	0.00189	0.127	
2023	AO-PIM-$1_{80\%}$|cPIM-$1_{20\%}$	2,6-DPPAQ/ $K_4Fe(CN)_6$	0.1/0.1	100	53	90	1800 (192 h)	0.000750	0.168	42
**2023**	**MTCP-50**	**MV/ TEMPTMA**	**2.0/2.0**	**100**	**77.2**	**311.7**	**500 (500 h)**	**0.00262**	**0.062**	**This work**
			**1.5/1.5**	**100**	**77.8**	**294**	**215 (1140 h)**	**0.032**	**0.145**	

[a] Inferred from graph in paper.

[a,b] Calculated from the reported experimental conditions (cell capacity, electrode area, cycling current density, etc.) and the capacity retention per cycle.

[c] Calculated from the capacity fade rate.

Supplementary Table 6. Summary and comparison of representative AEMWEs performance and stability.

Year	Membrane	A/C Ionomer	A/C Catalyst	Metal loading (mg cm^{-2})	A/C Electrolyte	Temperature (°C)	Voltage (V)	Current density (A cm^{-2})	Energy efficiency (%)	Current density (A cm^{-2})	Durability (h)	Voltage decay rate (mV h^{-1})	Reference
2012	A201	AS-4	IrO_2/ Pt/C	2.9/2.0	1 M KOH	50	1.8	0.399	NA	0.2	535	0.74	43
2012	xQAPS	xQAPS	Ni-Fe/ Ni-Mo	NA	Pure water	70	1.8	0.24	NA	NA			44
2018	TPN1-100	AS-4	PGM	2.0/2.0	1 M KOH	50	1.95	0.1	NA	0.2	5.83	12	45
2019	PBI+KOH	NA	Ni/ NiMo	NA	24% KOH	80	1.8	1.8	84.6	NA			46
2019	PVBC-MPy/35% PEKcardo	NA	NiMo/ NiFe	0.25/ 0.5	1 M KOH	60	2	0.5	65.3	0.5	46	3.47	47
2020	QMter-co-Mpi-100%	QMter-co-Mpi-100%	IrO_2/ Pt/C	2.0/2.0	1 M KOH	50	1.8	0.15	59.5	0.2	500	0.2	48
2020	PTFESustainion®	Nafion	Ni-Mo/ Fe-Ni-Mo	NA	1 M KOH	80	1.57	1.0	91.0	NA			49
2020	AF1-HNN8-50-X	FAA	IrO_2/ NiFe	3.5/1.0	1 M KOH	50	1.8	1.44	82.6	0.5	16.6	2.39	50
2020	HTMA-DAPP	TMA-70	NiFe / PtRu/C	3.0/2.0	1 M NaOH	60	1.8	5.3	90.5	0.2	170	1.23	51
					Pure water	85	1.8	2.75	88.8	NA	NA	NA	

2020	QPC-TMA	QPC-TMA	IrO_2/ Pt/C	2.0/0.4	1 M KOH	70	1.8	2.5	88.2	NA	2.7	55% performance loss	8
					Pure water	70	1.8	0.2	NA	NA	NA	NA	
2021	PTFE/ LDH	NA	NiFe/ CoP	NA	1 M KOH	60	1.8	1.0	75.6	0.5	180	NA	52
2021	HWU-AEM	VBQPPO	NiFe/ PtRu/C	NA/1.0	1 M KOH	60	1.8	1.3	81.7	0.5	162	0.417	53
					Pure water	60	1.8	0.16	NA	NA			
2021	M-6#	NA	NiFe/ NiMo	NA	1 M KOH	80	1.8	0.4	66.5	0.5	168	0.59	54
2021	Sustainion x37 50	Sustainion XA-9	Acta 3030/ Acta 4030	2.16/ 2.16	Pure water	60	1.8	0.34	NA	0.5	170	0.81	55

2021	PAP-TP-85	PAP-TP-MQN	FexNiy OOH-20F	NA/ 0.94	1 M KOH	80	1.8	0.83	77.5	0.5	70	1.81	56
2021	PFTP-13	PFTP-8/ PFBP-14	IrO_2/ Pt/C	2.0/0.5	1 M KOH	80	1.8	3.8	88.8	0.5	1100	0.2	57
					Pure water		1.8	0.83	77.8	NA	NA	NA.	
			NiFe/ NiFe	20/20	1 M KOH		1.8	0.88	77.9	0.5	1000	No deg.	
2022	PQP-100-	PBP-67	IrO_2/ Pt/C	1.5/1.3	1 M NaOH	85	1.8	0.8	77.7%	0.2/60	402	0.11	58
2022	PBI	NA	VCoP/ VCoP	NA	1 M KOH	60	1.8	2.0	84.1%	1.0	200	0.05	59
	PDTP				Pure water			1.6	84.1%	1.0	600	0.1	
2022	QPP-B-PSK-3.5-	QPC-TMA	NiFe/ Pt/C	1.0/0.4	1 M KOH	90	1.8	3.15	90.5%	1.0	100	1.2	60

	TMA												
**2023**	**MTCP-50**	**MTCP-50-20%/MTCP-0%**	**NiFe/Pt/Ru/C**	**4.0/1.0**	**Pure water**	**60**	**1.8**	**0.82**	**77.1**	NA			**This work**
						**70**		**0.94**	**78.7**				
						**80**		**1.11**	**80.5**				
						**90**		**1.29**	**82.6**				
					**1 M KOH**	**60**		**4.0**	**91.6**	**0.5**	**2500**	**0.01456**	
						**70**		**4.68**	**92.2**				
						**80**		**5.0**	**94.0**	**1.0**	**500**	**0.13**	
						**90**		**5.4**	**94.7**				

Supplementary Table 7. Summary and comparison of representative AEMFCs performance.

**Year**	**Membrane**	**A/C Ionomer**	**Anode**	**Cathode**	**A/C Metal loading (mg cm^{-2})**	**A/C Fuel**	**Cell temperature (°C)**	**A/C Back pressure (MPa)**	**Power density (W cm^{-2})**	**Reference**
2018	ETFE-BTMA	ETFE-BTMA	PtRu/C	Pt/C	0.5/0.5	H_2/O_2	60	0	1.9	61

2018	QAPPT	QAPPT	Pt/C	PtRu/C	0.4/0.4	H_2/O_2	80	0.2	2.08	62
					0.1/0.4				1.66	
2018	TPN	FLN-55	PtRu/C	Pt/C	0.5/0.6	H_2/O_2	80	0.285	1.46	63
2019	HDPE	ETFE-BTMA	PtRu/C	Pt/C	0.4/0.4	H_2/O_2	80	0	2.55	64
2019	PAP-TP-85	PAP-BP-100/ PAP-TP-100	Pt/C	silver-based	0.15/1.0	H_2/air	95	0.25/0.13	0.92	20
2019	PX75-T50	Poly (vinylbenzyl chloride)	PtRu/C	Pt/C	0.5/0.5	H_2/O_2	80	0	0.73	19
2020	QPC-TMA	QPC-TMA	PtRu/C	Pt/C	0.4/0.4	H_2/O_2	60	0	1.61	8
2020	GT64-15	GT78/GT32	PtRu/C	Pt/C	0.7/0.6	H_2/O_2	80	NA	3.2	65
2020	PDTP-25	PFBP/PDTP-75	PtRu/C	Pt/C	0.39/0.26	H_2/O_2	NA	0.13	2.58	22
2021	ZIL-MEA	VBBQPPO-	PtRu/C	Pt/C	0.5/0.5	H_2/O_2	70	0.2	1.5	66
2021	PFTP-13	PFBP-14	PtRu/C	Pt/C	0.42/0.33	H_2/O_2	80	0.13	2.34	4
2021	b-PTP-2.5	PFBP	PtRu/C	Pt/C	0.6/0.4	H_2/O_2	80	0.13	2.3	23

2022	MM-LPF-OH	MM-LPF-OH	Pt/C	Pt/C	0.6/0.6	H_2/O_2	80	0	0.737	24
2022	Cr-QPPV-2.51	Cr-QPPV-x	PtRu/C	Pt/C	0.5/0.5	H_2/O_2	80	0	1.27	67
2023	FPAP-3	PFBP and PTFE	PtRu/C	Pt/C	0.7/0.7	H_2/O_2	80	0	2	25
**2023**	**MTCP-50**	**MTCP-50-20%/ MTCP-50-0%**	**PtRu/C**	**Pt/C**	**0.2/0.2**	**H_2/O_2**	**90**	**0.1**	**1.61**	**This work**

Supplementary Table 8. Gel content of MTCP-x AEMs

AEMs	**MTCP-30**	**MTCP-50**	**MTCP-70**
Gel content (%)	93.54±0.4	93.50±0.5	93.57±0.5

Notes: The gel content of the cross-linked MTCP-x AEMs were measured by immersing the thoroughly dried MTCP-x samples in NMP solution at 60 ºC for 1 h followed by drying and weighing. The gel content was calculated from the ratio of the dried sample weight after test to its initial weight.

Reference

1. Thompson KA, Mathias R, Kim D, Kim J, Rangnekar N, Johnson JR*, et al.* N-Aryl–linked spirocyclic polymers for membrane separations of complex hydrocarbon mixtures. *Science* **369**, 310-315 (2020).
2. Tang H, Geng K, Wu L, Liu J, Chen Z, You W*, et al.* Fuel cells with an operational range of – 20 °C to 200 °C enabled by phosphoric acid-doped intrinsically ultramicroporous membranes. *Nat. Energy* **7**, 153-162 (2022).
3. Tan R, Wang A, Malpass-Evans R, Williams R, Zhao EW, Liu T*, et al.* Hydrophilic microporous membranes for selective ion separation and flow-battery energy storage. *Nat. Mater.* **19**, 195-202 (2020).
4. Chen N, Wang HH, Kim SP, Kim HM, Lee WH, Hu C*, et al.* Poly(fluorenyl aryl piperidinium) membranes and ionomers for anion exchange membrane fuel cells. *Nat. Commun.* **12**, 2367 (2021).
5. Meek KM, Antunes CM, Strasser D, Owczarczyk ZR, Neyerlin A, Pivovar BS. High-Throughput Anion Exchange Membrane Characterization at NREL. *ECS Trans.* **92**, 723-731 (2019).
6. Olsson JS, Pham TH, Jannasch P. Poly(arylene piperidinium) Hydroxide Ion Exchange Membranes: Synthesis, Alkaline Stability, and Conductivity. *Adv. Funct. Mater.* 2018, **28**, 1702758 (2018).
7. Chen N, Jin Y, Liu H, Hu C, Wu B, Xu S*, et al.* Insight into the Alkaline Stability of N-Heterocyclic Ammonium Groups for Anion-Exchange Polyelectrolytes. *Angew. Chem. Int. Ed. Engl.* **60**, 19272-19280 (2021).
8. Cha MS, Park JE, Kim S, Han S-H, Shin S-H, Yang SH*, et al.* Poly(carbazole)-based anion-conducting materials with high performance and durability for energy conversion devices. *Energy Environ. Sci.* **13**, 3633-3645 (2020).
9. Stephens PJ, Devlin FJ, Chabalowski CF, Frisch MJ. Ab Initio Calculation of Vibrational Absorption and Circular Dichroism Spectra Using Density Functional Force Fields. *J. Phys. Chem.* **98**, 11623-11627 (1994).
10. Mohanty AD, Ryu CY, Kim YS, Bae C. Stable Elastomeric Anion Exchange Membranes Based on Quaternary Ammonium-Tethered Polystyrene-b-poly(ethylene-co-butylene)-b-polystyrene Triblock Copolymers. *Macromolecules* **48**, 7085-7095 (2015).

11. Lee WH, Park EJ, Han J, Shin DW, Kim YS, Bae C. Poly(terphenylene) Anion Exchange Membranes: The Effect of Backbone Structure on Morphology and Membrane Property. *ACS Macro. Lett.*, **6**, 566-570 (2017).
12. Lee KH, Cho DH, Kim YM, Moon SJ, Seong JG, Shin DW*, et al.* Highly conductive and durable poly(arylene ether sulfone) anion exchange membrane with end-group cross-linking. *Energy Environ. Sci.* **10**, 275-285 (2017).
13. Wang L, Brink JJ, Liu Y, Herring AM, Ponce-González J, Whelligan DK*, et al.* Non-fluorinated pre-irradiation-grafted (peroxidated) LDPE-based anion-exchange membranes with high performance and stability. *Energy Environ. Sci.* **10**, 2154-2167 (2017).
14. Pham TH, Olsson JS, Jannasch P. N-Spirocyclic Quaternary Ammonium Ionenes for Anion-Exchange Membranes. *J. Am. Chem. Soc.* **139**, 2888-2891 (2017).
15. Chen N, Long C, Li Y, Wang D, Zhu H. High-performance layered double hydroxide/poly(2,6-dimethyl-1,4-phenylene oxide) membrane with porous sandwich structure for anion exchange membrane fuel cell applications. *J. Membr. Sci.* **552**, 51-60 (2018).
16. Ahmed Mahmoud AM, Miyatake K. Optimization of the pendant chain length in partially fluorinated aromatic anion exchange membranes for alkaline fuel cells. *J. Mater. Chem. A* **6**, 14400-14409 (2018).
17. Peng H, Li Q, Hu M, Xiao L, Lu J, Zhuang L. Alkaline polymer electrolyte fuel cells stably working at 80 °C. *J. Power Sources* **390**, 165-167 (2018).
18. Zhu L, Peng X, Shang SL, Kwasny MT, Zimudzi TJ, Yu X*, et al.* High Performance Anion Exchange Membrane Fuel Cells Enabled by Fluoropoly(olefin) Membranes. *Adv. Funct. Mater.* **29**, 1902059 (2019).
19. Kim Y, Wang Y, France-Lanord A, Wang Y, Wu YM, Lin S*, et al.* Ionic Highways from Covalent Assembly in Highly Conducting and Stable Anion Exchange Membrane Fuel Cells. *J. Am. Chem. Soc.* **141**, 18152-18159 (2019).
20. Wang J, Zhao Y, Setzler BP, Rojas-Carbonell S, Ben Yehuda C, Amel A*, et al.* Poly(aryl piperidinium) membranes and ionomers for hydroxide exchange membrane fuel cells. *Nat. Energy* **4**, 392-398 (2019).
21. Mandal M, Huang G, Hassan NU, Mustain WE, Kohl PA. Poly(norbornene) anion conductive membranes: homopolymer, block copolymer and random copolymer properties and performance. *J. Mater. Chem. A* **8**, 17568-17578 (2020).
22. Chen N, Hu C, Wang HH, Kim SP, Kim HM, Lee WH*, et al.* Poly(Alkyl-Terphenyl Piperidinium) Ionomers and Membranes with an Outstanding Alkaline-Membrane Fuel-Cell Performance of 2.58 W cm(-2). *Angew. Chem. Int. Ed. Engl.* **60**, 7710-7718 (2021).
23. Wu X, Chen N, Klok HA, Lee YM, Hu X. Branched Poly(Aryl Piperidinium) Membranes for Anion-Exchange Membrane Fuel Cells. *Angew. Chem. Int. Ed.* **61**, e202114892 (2022).
24. Liu X, Xie N, Xue J, Li M, Zheng C, Zhang J*, et al.* Magnetic-field-oriented mixed-valence-stabilized ferrocenium anion-exchange membranes for fuel cells. *Nat. Energy* **7**, 329-339 (2022).
25. Wu X, Chen N, Hu C, Klok H-A, Lee YM, Hu X. Fluorinated Poly(Aryl Piperidinium) Membranes for Anion Exchange Membrane Fuel Cells. *Adv. Mater.* 2210432 (2023).
26. Janoschka T, Martin N, Hager MD, Schubert US. An Aqueous Redox-Flow Battery with High Capacity and Power: The TEMPTMA/MV System. *Angew. Chem. Int. Ed. Engl.* **55**, 14427-14430 (2016).

27. Beh ES, De Porcellinis D, Gracia RL, Xia KT, Gordon RG, Aziz MJ. A Neutral pH Aqueous Organic–Organometallic Redox Flow Battery with Extremely High Capacity Retention. *ACS Energy Lett.* **2**, 639-644 (2017).
28. DeBruler C, Hu B, Moss J, Liu X, Luo J, Sun Y*, et al.* Designer Two-Electron Storage Viologen Anolyte Materials for Neutral Aqueous Organic Redox Flow Batteries. *Chem* **3**, 961-978 (2017).
29. Hu B, DeBruler C, Rhodes Z, Liu TL. Long-Cycling Aqueous Organic Redox Flow Battery (AORFB) toward Sustainable and Safe Energy Storage. *J. Am. Chem. Soc.* **139**, 1207-1214 (2017).
30. Luo J, Hu B, Debruler C, Liu TL. A π-Conjugation Extended Viologen as a Two-Electron Storage Anolyte for Total Organic Aqueous Redox Flow Batteries. *Angew. Chem. Int. Ed.* **57**, 231-235 (2018).
31. Liu Y, Goulet M-A, Tong L, Liu Y, Ji Y, Wu L*, et al.* A Long-Lifetime All-Organic Aqueous Flow Battery Utilizing TMAP-TEMPO Radical. *Chem* **5**, 1861-1870 (2019).
32. Huang Z, Kay CWM, Kuttich B, Rauber D, Kraus T, Li H*, et al.* An "interaction-mediating" strategy towards enhanced solubility and redox properties of organics for aqueous flow batteries. *Nano Energy* **69**, 104464 (2020).
33. Hu S, Li T, Huang M, Huang J, Li W, Wang L*, et al.* Phenylene-Bridged Bispyridinium with High Capacity and Stability for Aqueous Flow Batteries. *Adv. Mater.* **33**, e2005839 (2021).
34. Hu B, Fan H, Li H, Ravivarma M, Song J. Five-Membered Ring Nitroxide Radical: A New Class of High-Potential, Stable Catholytes for Neutral Aqueous Organic Redox Flow Batteries. *Adv. Funct. Mater.* **31**, 2102734 (2021).
35. Hu B, Hu M, Luo J, Liu TL. A Stable, Low Permeable TEMPO Catholyte for Aqueous Total Organic Redox Flow Batteries. *Adv. Energy Mater.* **12**, 2102577 (2021).
36. Pan M, Gao L, Liang J, Zhang P, Lu S, Lu Y*, et al.* Reversible Redox Chemistry in Pyrrolidinium-Based TEMPO Radical and Extended Viologen for High-Voltage and Long-Life Aqueous Redox Flow Batteries. *Adv. Energy Mater.* **12**, 2103478 (2022).
37. Luo J, Hu M, Wu W, Yuan B, Liu TL. Mechanistic insights of cycling stability of ferrocene catholytes in aqueous redox flow batteries. *Energy Environ. Sci.* **15**, 1315-1324 (2022).
38. Luo J, Hu B, Debruler C, Bi Y, Zhao Y, Yuan B*, et al.* Unprecedented Capacity and Stability of Ammonium Ferrocyanide Catholyte in pH Neutral Aqueous Redox Flow Batteries. *Joule* **3**, 149-163 (2019).
39. Zuo P, Li Y, Wang A, Tan R, Liu Y, Liang X*, et al.* Sulfonated Microporous Polymer Membranes with Fast and Selective Ion Transport for Electrochemical Energy Conversion and Storage. *Angew. Chem. Int. Ed. Engl.* **59**, 9564-9573 (2020).
40. Li X, Gao P, Lai Y-Y, Bazak JD, Hollas A, Lin H-Y*, et al.* Symmetry-breaking design of an organic iron complex catholyte for a long cyclability aqueous organic redox flow battery. *Nat. Energy* **6**, 873-881 (2021).
41. Ye C, Wang A, Breakwell C, Tan R, Grazia Bezzu C, Hunter-Sellars E*, et al.* Development of efficient aqueous organic redox flow batteries using ion-sieving sulfonated polymer membranes. *Nat. Commun.* **13**, 3184 (2022).
42. Wang A, Tan R, Liu D, Lu J, Wei X, Alvarez-Fernandez A*, et al.* Ion-Selective Microporous Polymer Membranes with Hydrogen-Bond and Salt-Bridge Networks for Aqueous Organic Redox Flow Batteries. *Adv. Mater.* e2210098 (2023).

43. Leng Y, Chen G, Mendoza AJ, Tighe TB, Hickner MA, Wang CY. Solid-state water electrolysis with an alkaline membrane. *J. Am. Chem. Soc.* **134**, 9054-9057 (2012).
44. Xiao L, Zhang S, Pan J, Yang C, He M, Zhuang L*, et al.* First implementation of alkaline polymer electrolyte water electrolysis working only with pure water. *Energy Environ. Sci.* **5,** 7869-7871 (2012).
45. Park EJ, Capuano CB, Ayers KE, Bae C. Chemically durable polymer electrolytes for solid-state alkaline water electrolysis. *J. Power Sources* **375,** 367-372 (2018).
46. Kraglund MR, Carmo M, Schiller G, Ansar SA, Aili D, Christensen E*, et al.* Ion-solvating membranes as a new approach towards high rate alkaline electrolyzers. *Energy Environ. Sci.* **12**, 3313-3318 (2019).
47. Li H, Kraglund MR, Reumert AK, Ren X, Aili D, Yang J. Poly(vinyl benzyl methylpyrrolidinium) hydroxide derived anion exchange membranes for water electrolysis. *J. Mater. Chem. A* **7**, 17914-17922 (2019).
48. Yan X, Yang X, Su X, Gao L, Zhao J, Hu L*, et al.* Twisted ether-free polymer based alkaline membrane for high-performance water electrolysis. *J. Power Sources* **480**, 228805 (2020).
49. Chen P, Hu X. High-Efficiency Anion Exchange Membrane Water Electrolysis Employing Non-Noble Metal Catalysts. *Adv. Energy Mater.* **10**, 2002285 (2020).
50. Fortin P, Khoza T, Cao X, Martinsen SY, Oyarce Barnett A, Holdcroft S. High-performance alkaline water electrolysis using Aemion™ anion exchange membranes. *J. Power Sources* **451**, 227814 (2020).
51. Li D, Park EJ, Zhu W, Shi Q, Zhou Y, Tian H*, et al.* Highly quaternized polystyrene ionomers for high performance anion exchange membrane water electrolysers. *Nat. Energy* **5**, 378-385 (2020).
52. Wan L, Xu Z, Wang B. Green preparation of highly alkali-resistant PTFE composite membranes for advanced alkaline water electrolysis. *Chem. Eng. J.* **426**, 131340 (2021).
53. Xu Z, Wan L, Liao Y, Wang P, Liu K, Wang B. Anisotropic anion exchange membranes with extremely high water uptake for water electrolysis and fuel cells. *J. Mater. Chem. A* **9**, 23485-23496 (2021).
54. Li H, Yu N, Gellrich F, Reumert AK, Kraglund MR, Dong J*, et al.* Diamine crosslinked anion exchange membranes based on poly(vinyl benzyl methylpyrrolidinium) for alkaline water electrolysis. *J. Membr. Sci.* **633**, 119418 (2021).
55. Razmjooei F, Morawietz T, Taghizadeh E, Hadjixenophontos E, Mues L, Gerle M*, et al.* Increasing the performance of an anion-exchange membrane electrolyzer operating in pure water with a nickel-based microporous layer. *Joule* **5**, 1776-1799 (2021).
56. Xiao J, Oliveira AM, Wang L, Zhao Y, Wang T, Wang J*, et al.* Water-Fed Hydroxide Exchange Membrane Electrolyzer Enabled by a Fluoride-Incorporated Nickel–Iron Oxyhydroxide Oxygen Evolution Electrode. *ACS Catal.* **11**, 264-270 (2020).
57. Chen N, Paek SY, Lee JY, Park JH, Lee SY, Lee YM. High-performance anion exchange membrane water electrolyzers with a current density of 7.68 A cm−2 and a durability of 1000 hours. *Energy Environ. Sci.* **14**, 6338-6348 (2021).
58. Liu M, Hu X, Hu B, Liu L, Li N. Soluble poly(aryl piperidinium) with extended aromatic segments as anion exchange membranes for alkaline fuel cells and water electrolysis. *J. Membr. Sci.* **642**, 119966 (2022).
59. Wan L, Xu Z, Xu Q, Wang P, Wang B. Overall design of novel 3D-ordered MEA with

drastically enhanced mass transport for alkaline electrolyzers. *Energy Environ. Sci.* **15**, 1882-1892 (2022).

60. Cha MS, Park JE, Kim S, Shin S-H, Yang SH, Lee SJ*, et al.* Oligomeric chain extender-derived anion conducting membrane materials with poly(p-phenylene)-based architecture for fuel cells and water electrolyzers. *J. Mater. Chem. A* **10**, 9693-9706 (2022).
61. Omasta TJ, Park AM, LaManna JM, Zhang Y, Peng X, Wang L*, et al.* Beyond catalysis and membranes: visualizing and solving the challenge of electrode water accumulation and flooding in AEMFCs. *Energy Environ. Sci.* **11**, 551-558 (2018).
62. Li Q, Peng H, Wang Y, Xiao L, Lu J, Zhuang L. The Comparability of Pt to Pt-Ru in Catalyzing the Hydrogen Oxidation Reaction for Alkaline Polymer Electrolyte Fuel Cells Operated at 80 degrees C. *Angew. Chem. Int. Ed. Engl.* **58**, 1442-1446 (2019).
63. Maurya S, Noh S, Matanovic I, Park EJ, Narvaez Villarrubia C, Martinez U*, et al.* Rational design of polyaromatic ionomers for alkaline membrane fuel cells with >1 W cm−2 power density. *Energy Environ. Sci.* **11**, 3283-3291 (2018).
64. Wang L, Peng X, Mustain WE, Varcoe JR. Radiation-grafted anion-exchange membranes: the switch from low- to high-density polyethylene leads to remarkably enhanced fuel cell performance. *Energy Environ. Sci.* **12**, 1575-1579 (2019).
65. Ul Hassan N, Mandal M, Huang G, Firouzjaie HA, Kohl PA, Mustain WE. Achieving High-Performance and 2000 h Stability in Anion Exchange Membrane Fuel Cells by Manipulating Ionomer Properties and Electrode Optimization. *Adv. Energy Mater.* **10**, 2001986 (2020).
66. Liang X, Ge X, He Y, Xu M, Shehzad MA, Sheng F*, et al.* 3D-Zipped Interface: In Situ Covalent-Locking for High Performance of Anion Exchange Membrane Fuel Cells. *Adv. Sci.* **8**, 2102637 (2021).
67. Zhang F, Zhang Y, Sun L, Wei C, Zhang H, Wu L*, et al.* A pi-Conjugated Anion-Exchange Membrane with an Ordered Ion-Conducting Channel via the McMurray Coupling Reaction. *Angew. Chem. Int. Ed. Engl.* **62**, e202215017 (2023).
