# Supplementary material for: Upscaled production of an ultramicroporous anion-exchange membrane enables long-term operation in electrochemical energy devices
Source: Nat Commun. 2023 May 12;14:2732. doi: 10.1038/s41467-023-38350-7 (PMC10175247; doi:10.1038/s41467-023-38350-7)
Supplement: Supplementary file 3 — Description of Additional Supplementary Files [file 41467_2023_38350_MOESM3_ESM.docx]

**Description of Additional Supplementary Files**

**Supplementary Video 1.** Large-scale preparation video of AEMs.

**Supplementary Video 1.** Roll to roll production video of AEMs.
